# A Review of *ε*-Ga_2_O_3_ Films: Fabrications and Photoelectric Properties

**DOI:** 10.3390/ma18112630

**Published:** 2025-06-04

**Authors:** Siwei Wang, Jie Jian, Cong Xu, Xiaoheng Dong, Jielong Yang, Maolin Zou, Wangwang Liu, Qinglong Tu, Mengyao Li, Cheng Cao, Xiangli Liu

**Affiliations:** 1 School of Materials Science and Engineering, Harbin Institute of Technology (Shenzhen), Shenzhen 518055, China; 24b955018@stu.hit.edu.cn (S.W.); 22s155085@stu.hit.edu.cn (X.D.); 24s155046@stu.hit.edu.cn (W.L.); 23s155098@stu.hit.edu.cn (Q.T.); 23s155106@stu.hit.edu.cn (M.L.); 2 Institute of Special Environments Physical Sciences, Harbin Institute of Technology (Shenzhen), Shenzhen 518055, China; jianjie@hit.edu.cn (J.J.); 22s155081@stu.hit.edu.cn (C.X.); 22s155040@stu.hit.edu.cn (J.Y.); 23s155006@stu.hit.edu.cn (M.Z.); 24s155077@stu.hit.edu.cn (C.C.)

**Keywords:** *ε*-Ga_2_O_3_, film fabrications, physical vapor deposition, chemical vapor deposition, microstructure, photoelectric properties

## Abstract

Gallium oxide (Ga_2_O_3_), as an ultra-wide bandgap semiconducting material, has attracted extensive research interest in recent years. Owing to its outstanding electrical and optical properties, as well as its high reliability, Ga_2_O_3_ shows great potential in power electronics, optoelectronics, memory devices, and so on. Among all the different polymorphs, *ε*-Ga_2_O_3_ is the second most thermally stable phase. It has a hexagonal crystal structure, which contributes to its isotropic physical properties and its suitable growth on low-cost commercial substrates, such as Al_2_O_3_, Si (111). However, there are far fewer research works on *ε*-Ga_2_O_3_ in comparison with the most thermally stable β phase. Aiming to provide a comprehensive view on the current works of *ε*-Ga_2_O_3_ and support future research, this review conducts detailed summarizations for the fabrication processes of *ε*-Ga_2_O_3_ thin films and the photoelectrical properties of *ε*-Ga_2_O_3_-based photodetectors. The effects of different deposition parameters on film phases and qualities are discussed. The forming mechanisms of *ε* phase prepared by chemical vapor depositions (CVDs) and physical vapor depositions (PVDs) are analyzed, respectively. Conclusions are made concerning the relationships between film microstructures and properties. In addition, strategies for further improving *ε*-Ga_2_O_3_ film performance are briefly summarized.

## 1. Introduction

Since the 1950s, semiconductor materials have been widely used in computer microprocessors [1,2,3], solar cells [4,5], and power amplifiers [6,7]. Silicon, as a first-generation semiconductor material, is most commonly used to manufacture devices such as diodes and transistors for integrated circuit applications. Due to its low critical electric field strength (Ec) [8], the performance of silicon-based power devices has reached its peak. For power switch applications, low Ec limits operating voltage capability. Simultaneously, the narrow bandgap and low breakdown voltage result in a performance ceiling for silicon RF devices limited by power frequency products. These characteristics cannot be overcome by traditional preparation methods. Therefore, semiconductor materials with innate performance advantages have attracted significant attention in recent decades. With significant advantages in reducing energy consumption in power transmission and having an efficient light absorption capacity in the ultraviolet band, wide bandgap materials shine in the fields of power devices, radio frequency devices, and photoelectric detection [8,9].

Gallium oxide (Ga_2_O_3_) is an ultra-wide bandgap semiconductor material, as shown in Table 1, with a bandgap of 4.7–5.2 eV [10,11]. Compared with silicon carbide (SiC) materials, the commercially used third-generation semiconductor material, Ga_2_O_3_ has more than three times higher insulation breakdown field strength and lower background carrier concentration. In power electronics applications, it can achieve higher voltage resistance, smaller device volume, better doping controllability, and faster switching speeds [12,13,14,15,16,17,18,19]. At the same time, Ga_2_O_3_ demonstrates higher chemical and thermal stability, making it more suitable for high temperature, irradiation, and other extreme environmental applications. In addition, its ultra-wide bandgap makes this semiconductor suitable for manufacturing solar-blind detectors for UV-C radiation in the wavelength range from 200 nm to 280 nm.

As early as 1952, Roy et al. [20] has reported five polymorphs (*α*, *β*, *ε*, *γ*, *δ*) of gallium oxide (Ga_2_O_3_), of which *β* crystal is the most thermodynamically stable form. Other crystal structure forms tend to transform into *β* at elevated temperatures [21,22]. *β*-Ga_2_O_3_ belongs to the monoclinic crystal system with a space group of C2/m, each cell is composed of two GaO_4_ tetrahedrons and two GaO_6_ octahedrons. The tetrahedral GaO_4_ chemical bond length ranges from 1.80 Å to 1.85 Å and the bond length of the octahedral GaO_6_ is from 1.95 Å to 2.08 Å. Because of its thermodynamic stability, most research has been concentrated on the *β*-Ga_2_O_3_ phase. Efforts have been focused on the fabrication of *β*-Ga_2_O_3_ wafers, the homoepitaxial growth of *β*-Ga_2_O_3_ films, and the power device applications, as well as the improvement of photoelectronic performance in the solar-blind waveband.

Owing to its excellent intrinsic properties, such as ultra-wide bandwidth, ultra-high breakdown field strength, etc., and its ability to be grown from the melt, Ga_2_O_3_ has attracted enormous academic and industrial interest. The growth of large-size *β*-Ga_2_O_3_ crystal ingots has been reported via the “Czochralski method” [23,24] and the “edge-defined film-fed method” [25,26,27]. However, there have been no reports about successful growth of *ε*-Ga_2_O_3_ ingots or substrates, so far. Subsequently, hetero-epitaxial growth tends to be the most ideal approach for obtaining ε-Ga_2_O_3_ films, so it is a novel and valuable research topic in the academic community at present.

Galazka [23] has achieved the high-quality growth of single-crystal *β*-Ga_2_O_3_ bulk via the Czochralski method. The researcher achieved the growth of large-sized *β*-Ga_2_O_3_ with a 2-inch diameter by developing a specific oxygen supply scheme to regulate the growth process. The X-ray rocking curve full width at half maximum (FWHM) of the *β*-Ga_2_O_3_ bulk crystals is below 40 arcsec, with the length ≥ 6 cm and up to 2.5 cm for electrically insulating and highly conducting crystals.

High-quality *β*-Ga_2_O_3_ films have been successfully grown on various substrates and high-performance photodetectors have been fabricated. Arora et al. [28] fabricated *β*-Ga_2_O_3_-based self-powered photodetectors by depositing *β*-Ga_2_O_3_ on p-Si (100). The devices, which were prepared by introducing a high-temperature seed layer, had very low dark current (1.4 pA) and a high on/off ratio (>10^3^). Nie et al. [29] prepared a photoconductive photodetector with an extremely low dark current (0.4 pA) by magnetron sputtering. The shallow donors from gallium interstitials were suppressed by modulating their formation energy and promoting the generation of 2V_Ga_-Ga_i_, leading to the achievement of an ultralow dark current.

*ε*-Ga_2_O_3_ is the second most stable phase. It is commonly considered as a hexagonal structure (symmetry group P6_3_mc). The distinction between the *κ* and *ε* phases of Ga_2_O_3_ has been a longstanding topic. Recent research has suggested that *ε*-Ga_2_O_3_ possesses a pseudo-hexagonal crystal structure, resulting from in-plane 120° rotational nano-domains of orthorhombic *κ*-Ga_2_O_3_ (symmetry group Pna2_1_). In contrast, earlier studies could only observe the average disordered pseudo-hexagonal structure due to the limited resolution of TEM. XRD spectra of *ε*-Ga_2_O_3_ can be considered to the superimposition of the diffraction contributions generated by three orthorhombic lattices, rotated by 120° with respect to each other [10,30]. Nevertheless, this evidence remains insufficient for drawing definitive conclusions. This review will consider these two phases differently and mainly focus on the *ε*-Ga_2_O_3_.

Compared to *β*-Ga_2_O_3_, *ε*-Ga_2_O_3_ has a hexagonal structure, which contributes to enhanced epitaxial growth compatibility with low-cost commercial hexagonal-structured substrates, such as Al_2_O_3_ and SiC. Moreover, among all crystalline of Ga_2_O_3_, only *ε*-Ga_2_O_3_ shows strong spontaneous polarizability [31,32,33]. Benefiting from its strong spontaneous polarization, *ε*-Ga_2_O_3_ can potentially form two-dimensional electron gas with high mobility in heterojunction, which is helpful for solving the problem posed by the low electron mobility of the Ga_2_O_3_ material. The electron scattering of *ε*-Ga_2_O_3_ will benefit slightly from a higher forbidden bandwidth. The impurities inside *ε*-Ga_2_O_3_ are mostly deep-energy-level impurities, leading to a lower current in the dark state, which is more suitable for photodetectors. However, there is a paucity in the literature of ε-Ga_2_O_3_ studies, with publications accounting for only 5% of the *β*-Ga_2_O_3_ research articles. Most studies of *ε*-Ga_2_O_3_ focus on the synthesis conditions of the stable phase and the improvement of the electro-optical properties. Currently, the fundamental issues pertaining to the stable-film-forming mechanism of *ε*-Ga_2_O_3_ persist, and the reported photoelectronic properties vary a lot.

This review will mainly focus on analyzing the effects of various growth parameters for *ε*-Ga_2_O_3_ films synthesized by both chemical vapor deposition (CVD) and physical vapor deposition (PVD). And, by systematically comparing different growth conditions and deposition methods, the growth mechanisms of stable *ε*-Ga_2_O_3_ films will be summarized. Furthermore, the photoelectronic properties of *ε*-Ga_2_O_3_ films will be reviewed and the corresponding impact factors will be analyzed. This review provides a comprehensive view of the fabrications and properties of *ε*-Ga_2_O_3_ films, as well as their connections, which can give important guidance for further improvements in the performances of *ε*-Ga_2_O_3_ films, and studies on synthesizing them for other purposes.

## 2. Methods and Effects of Growth Parameters for *ε*-Ga_2_O_3_ Film Preparation

For the epitaxial growth of *ε*-Ga_2_O_3_ films, both the PVD and CVD techniques are suitable. Each method shows unique advantages but also has drawbacks due to different growth mechanisms. In the case of CVD, the gas-phase reaction fills the chamber, leading to the more uniform deposition of complex-shaped surfaces. CVD techniques also have relatively higher growth rates, making them suitable for depositing thick (in μm range) *ε*-Ga_2_O_3_ films. However, it is difficult to control impurities in the films due to the participation of complex chemical precursors. In the case of PVD, impurity levels can be maintained at a relatively low level due to simpler reaction sources. Moreover, PVD processes are more environmentally friendly, owing to their significantly fewer toxic exhaust gases. Whereas, the PVD processes usually have much lower reaction rates and result in slower growth rates, which makes them more suitable for depositing thin *ε*-Ga_2_O_3_ films. In addition, the reactants in PVD processes have difficulty reaching shadowed areas. Thus, PVD techniques are not suitable for depositing *ε*-Ga_2_O_3_ on complex surfaces, such as devices with trench structures.

As illustrated in Table 2, the characteristics of *ε*-Ga_2_O_3_ grown by CVD, including metal–organic chemical vapor deposition (MOCVD) and mist–chemical vapor deposition (Mist-CVD), are compared in terms of growth conditions, such as the precursor and growth temperature range. Furthermore, the characteristics of *ε*-Ga_2_O_3_ prepared by PVD, including pulsed-laser deposition (PLD) and molecular beam epitaxy (MBE), are reviewed in Table 3 in terms of their preparation conditions and growth kinetics. Accordingly, the effects of different parameters on the growth of *ε*-Ga_2_O_3_ will be investigated by analyzing CVD and PVD methods separately.

### 2.1. Chemical Vapor Deposition Method

In the current research on the growth of *ε*-Ga_2_O_3_ by CVD, the crucial parameters affecting the growth of thin films include growth temperature, growth pressure, precursor ratio, and substrate. Substrates exhibiting lower lattice mismatch with Ga_2_O_3_ enable higher-quality films growth. Growth temperature serves as a key factor in determining the phase composition in CVD technique. The optimal growth temperature for Ga_2_O_3_ is within a medium range; higher temperatures will promote the nucleation and growth of *β* phase, while lower temperatures result in amorphous films. Growth pressure is also important in controlling the final film phase, where lower pressure will favor the growth of *ε* phase, but there is still an optimal growth pressure range. Precursor ratio also modulates the phase composition of the final film. Both low gallium flux and high oxygen flux enhance the formation of the *ε* phase, and the precursors that provide strong oxidation can promote the formation of the *β* phase with relatively little flux. The specific role of each growth parameter effects the film phase will be described in this section.

#### 2.1.1. Substrate

The substrate provides the fundamental basis for epitaxy growth. The essence of epitaxy is the migration and reaction of particles in the gas phase on the substrate. The crystal structure of the substrate plays a critical role in determining the polymorphs and crystallinity of epitaxy films. In general, smaller lattice mismatch between film and substrate correlates with enhanced epitaxial film quality. However, controlling the lattice mismatch in certain ranges will allow a strain tuning of film, leading to new structures and properties. For materials with multiple polymorphs, such as Ga_2_O_3_, selecting substrates with tailored structures can initiate an easier growth of specific polymorph via lowering its formation energy. Therefore, the substrate effects for *ε*-Ga_2_O_3_ growth will be reviewed first.

It has been demonstrated that *ε*-Ga_2_O_3_ can be grown on many types of single-crystal substrates, including MgO (111) [49], yttria-stabilized ZrO_2_ (YSZ) (111) [49] and (100) [65], 3C-SiC (111) and (001) [38], 4H-SiC (0001) [66,67], 6H-SiC (0001) [68], AlN (0001) [69,70], GaN (0001) [69,71,72], Al_2_O_3_ (0001), etc., which mostly have cubic or hexagonal symmetry. This is because the cubic and hexagonal crystal structures exhibit a slight lattice mismatch with the structure of *ε*-Ga_2_O_3_. However, *ε*-Ga_2_O_3_ is not only grown on cubic or hexagonal substrates. It has been demonstrated that *ε*-Ga_2_O_3_ can also be epitaxially grown on the monoclinic *β*-Ga_2_O_3_ ($\overset{-}{2}$01) substrate [69].

**Figure 1:**
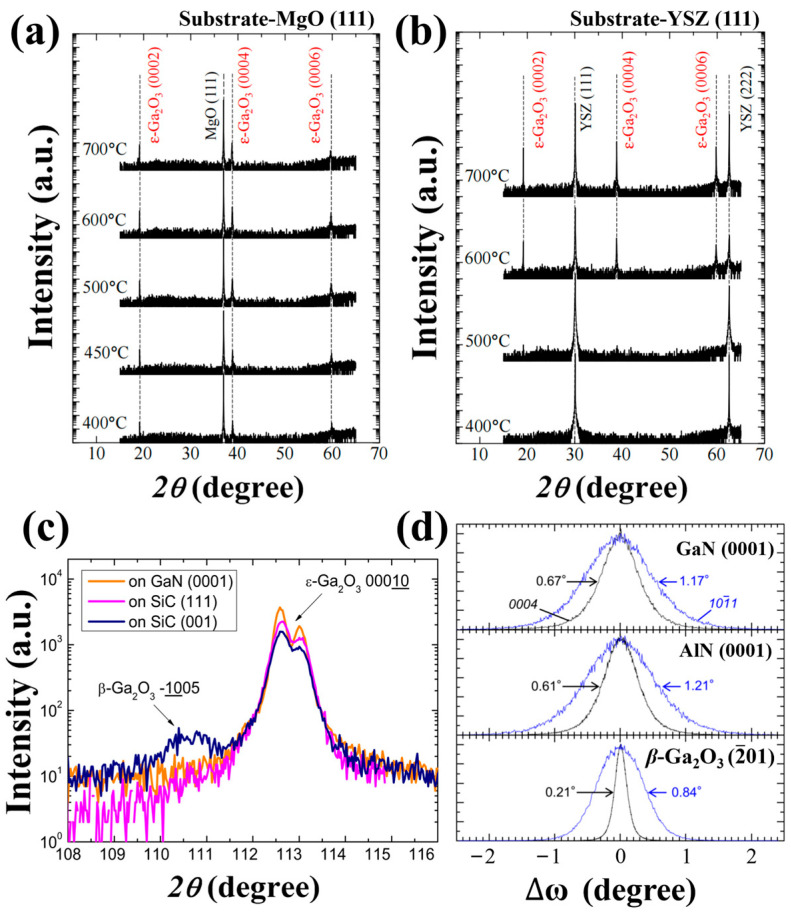
XRD 2θ-ω scan profiles of *ε*-Ga_2_O_3_ films grown on (**a**) MgO (111) substrate and (**b**) YSZ (111) substrate at different growth temperature [49] (reprinted from [49], with the permission of IOP Science 2016); (**c**) (00010) diffraction profiles of *ε*-Ga_2_O_3_ grown at 650 °C on different templates [38] (reprinted from [38], with the permission of Elsevier 2016); (**d**) XRCs of *ε*-Ga_2_O_3_ films grown on GaN (0001), AlN (0001), and *β*-Ga_2_O_3_ ($\overset{-}{2}$01) [69] (reprinted from [69], with the permission of AIP Publishing 2015).

Hiroyuki Nishinaka et al. [49] have epitaxially grown *ε*-Ga_2_O_3_ on MgO (111) and YSZ (111) substrates via Mist-CVD and explored the effect of temperature on epitaxial film growth. The XRD scan spectra of *ε*-Ga_2_O_3_ films are illustrated in Figure 1a,b. These spectra show that high-quality single *ε*-Ga_2_O_3_ can be grown on both MgO and YSZ substrates, indicating a high degree of fitness between *ε*-Ga_2_O_3_ and these substrates. Notably, the two substrates exhibit different epitaxial growth behaviors: *ε*-Ga_2_O_3_ can be grown on the MgO (111) substrate at low growth temperatures, whereas no growth occurs on YSZ (111) below 600 °C. This phenomenon originates from differences in lattice matching: MgO (111) has a smaller lattice mismatch with *ε*-Ga_2_O_3_ (001) compared to YSZ (111), resulting in the fact that, at low temperatures, MgO (111) will be more adapted to the growth of *ε*-Ga_2_O_3_.

Boschi, F et al. [38] studied the effects of same material substrates with different orientations on *ε*-Ga_2_O_3_. Figure 1c shows XRD scans of Ga_2_O_3_ grown on 3C-SiC (111), 3C-SiC (001) and GaN (0001) respectively. Among these three substrates, GaN (0001) has superior lattice matching compared to the others, which is attributed to its hexagonal symmetry. Although the 3C-SiC (111) substrate exhibits a high degree of cubic symmetry, it also shows considerable strength and sharpness in XRD spectra, given the hexagonal arrangement of the atoms in its specific plane. However, Ga_2_O_3_ grown on 3C-SiC (001) substrate shows diffraction peaks of *β*-Ga_2_O_3_-related crystal plane, *ε* phase is still the main phase.

Bhuiyan, A. F. M. et al. [65] grew *ε*-Ga_2_O_3_ on YSZ (100), AlN (0001), GaN (0001), and *c*-Al_2_O_3_ (0001) substrates. All the XRD spectra results showed significant *ε*-Ga_2_O_3_ diffraction peaks, indicating successful epitaxial growth on those different substrates. However, for *c*-Al_2_O_3_ (0001) substrate, in addition to the diffraction peaks of the *ε*-Ga_2_O_3_-related crystal plane, there are also diffraction peaks belonging to *β*-Ga_2_O_3_, demonstrating the critical role of substrate in controlling Ga_2_O_3_ epitaxial phase under identical growth conditions.

Oshima, Y et al. [69] studied the growth of *ε*-Ga_2_O_3_ on GaN (0001), AlN (0001), and *β*-Ga_2_O_3_ ($\overset{-}{2}$01) substrates. It was likewise demonstrated that the substrate has a great influence on the crystallization quality of epitaxial films. The FWHM comparisons in Figure 1d reveal that *ε*-Ga_2_O_3_ grown on *β*-Ga_2_O_3_ ($\overset{-}{2}$01) substrate shows a higher crystalline quality. Despite *β*-Ga_2_O_3_ does not have a hexagonal crystal structure similar to *ε*-Ga_2_O_3_, it may still have a lower lattice mismatch than GaN (0001) and AlN (0001). In addition to direct growth on single-crystal substrates, epitaxy can also be performed by introducing transition layer. Arata, Y. et al. [46] achieved the epitaxial of *ε*-Ga_2_O_3_ by inserting cubic NiO (111) on *c*-Al_2_O_3_ as a buffer layer, obtaining a smooth film with RMS roughness of 1.7 nm and a direct bandgap of about 4.9 eV.

**Figure 2:**
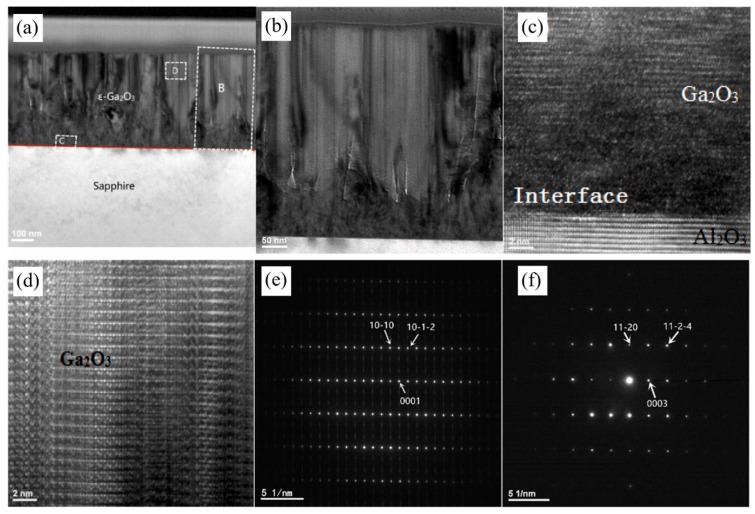
Cross-sectional TEM of the Ga_2_O_3_ thin film with thickness of 480 nm [35]. (**a**) Image of the whole film, (**b**–**d**) diagrams correspond to the dashed boxes B C D regions in (**a**), respectively; (**b**) magnified image of the region B indicated in (**a**); HRTEM micrograph of regions (**c**) C and (**d**) D indicated in (**a**); (**e**) SAED pattern of region D; (**f**) SAED pattern of sapphire substrate. Reprinted from [35], with the permission of Elsevier 2020.

To elucidate *ε*-Ga_2_O_3_ growth mechanisms and microstructure of *ε*-Ga_2_O_3_ on *c*-Al_2_O_3_, Cao et al. [31] conducted TEM characterizations on the 480 nm thick *ε*-Ga_2_O_3_ film, as shown in Figure 2. The TEM images of the cross-sections show a highly strained interface with numerous dislocations and grain boundaries between the film and the substrate. The highly distorted region is formed at the beginning of crystal growth, mainly due to a lattice mismatch of about 4.1% between *ε*-Ga_2_O_3_ and the *c*-Al_2_O_3_ substrate. However, the lattice structure of *ε*-Ga_2_O_3_ can be clearly seen in the film far away from the substrate, indicating that, under this condition, as the film thickness increases, the stress of the substrate on the film is gradually relieved and crystal quality gradually becomes stable [35]. The selective electron diffraction of Figure 2e proves the epitaxial relationships between *ε*-Ga_2_O_3_ and *c*-Al_2_O_3_, *ε*-Ga_2_O_3_ [10$\overset{-}{1}$0] ‖ *c*-Al_2_O_3_ [11$\overset{-}{2}$0]; this is also consistent with previous reports [31].

**Figure 3:**
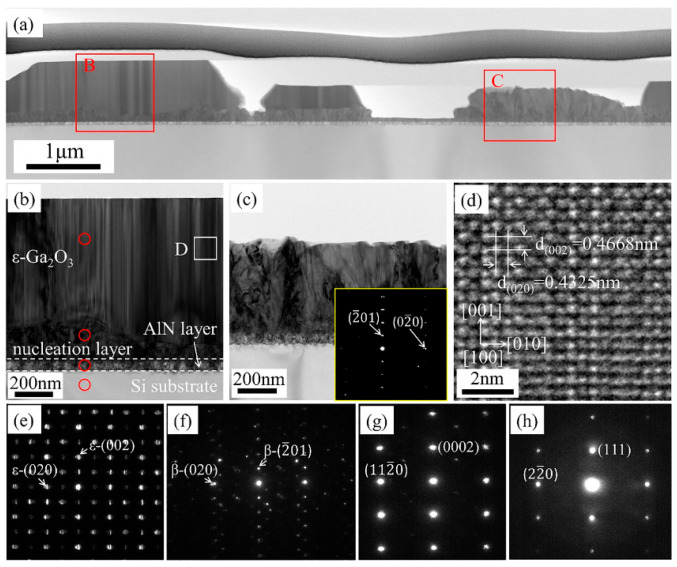
(**a**) Overview cross-sectional TEM image of *ε*-Ga_2_O_3_ on AlN/Si(111) template; (**b**,**c**) magnified TEM images marked by red square B and square C in (**a**), respectively, inset as shown in (**c**) indicates the electron diffraction pattern (EDP) of epilayer in region C; (**d**) magnified TEM image marked by white square D in (**b**); EDPs of (**e**) *ε*-Ga_2_O_3_ epilayer, (**f**) nucleation layer, (**g**) AlN layer and (**h**) Si substrate, respectively (red circles marked in (**b**)) [37]. Reprinted from [37], with the permission of Elsevier 2022.

TEM characterization of *ε*-Ga_2_O_3_ which was grown on AlN/Si(111) templates was conducted by Chen et al. [37]. The analysis results were illustrated in Figure 3. The cross-section view in Figure 3a shows that the *β*-Ga_2_O_3_ grains are comparable to or smaller than *ε*-Ga_2_O_3_ grains, suggesting phase competition between the two polymorphs. The existence of *β*-Ga_2_O_3_ will suppress the nucleation and growth of *ε*-Ga_2_O_3_ and impedes the formation and coalescence of films, and the crystallization quality of the nucleation layer is poor corresponding to Figure 3f.

The epitaxial growth of high-quality *ε*-Ga_2_O_3_ exhibits substrate versatility. The critical parameter of lattice mismatch coefficient significantly governs crystalline quality, as evidenced by the fact that the wider growth window for *ε*-Ga_2_O_3_ observed on hexagonal crystal system substrates compared to alternative. The lattice-matching relationship between common substrates and *ε*-Ga_2_O_3_ has been listed in Table 4. For the substrates with hexagonal crystal structures, (0001) planes are generally used for *ε*-Ga_2_O_3_ growth, with a lattice-matching relationship has a 90° rotation, i.e., *ε*-Ga_2_O_3_ (10$\overset{-}{1}$0)//Sub (11$\overset{-}{2}$0). For the substrates with cubic crystal structures, (111) planes with threefold symmetry are usually used. The lattice-matching relationship is that *ε*-Ga_2_O_3_ (11$\overset{-}{2}$0)//Sub (011). When the film and substrate have a very large misfit, the film tends to have a domain matching growth.

Nevertheless, substrate selection necessitates systematic evaluation based on specific application scenarios. For example, When the intrinsic properties of *ε*-Ga_2_O_3_ are only utilized for device development (such as photodetectors), high-quality crystal growth is the key to ensure the performance of the device, and a substrate with low lattice mismatch should be used (such as Al_2_O_3_ or SiC). According to Mezzadri, F’s study [31,32,33], *ε*-Ga_2_O_3_ shows strong ferro-electrical performance. In order to study its ferro-electrical properties, a conductive substrate is usually chosen as the bottom electrode, and the substrate can be chosen as conductive STO or aluminum-doped zinc oxide (AZO). When it is necessary to constitute a heterojunction with other materials for bandgap engineering control, choosing the corresponding substrate is critical; for example, the combination of *ε*-Ga_2_O_3_ with *ε*-Al_x_Ga_1-x_O_3_ is believed to generate high-density two-dimensional electron gas in phase interface. In summary, substrate optimization requires comprehensive consideration of both fundamental material properties (crystallographic compatibility, electrical characteristics) and application requirements (device architecture, heterostructure design). Therefore, researchers must perform multi-parameter evaluations balancing commercial viability with scientific objectives when selecting growth templates.

#### 2.1.2. Growth Temperature

The above studies indicate that high-quality growth of the *ε* phase can be achieved on diverse substrates. Substrate selection modifies the temperature window for *ε*-Ga_2_O_3_ deposition. However, it was found that the temperature dependences are similar for *ε*-Ga_2_O_3_ films grown on different substrates. Therefore, the temperature effects will be discussed for the films grown on *c*-Al_2_O_3_ as a representative in this section.

**Figure 4:**
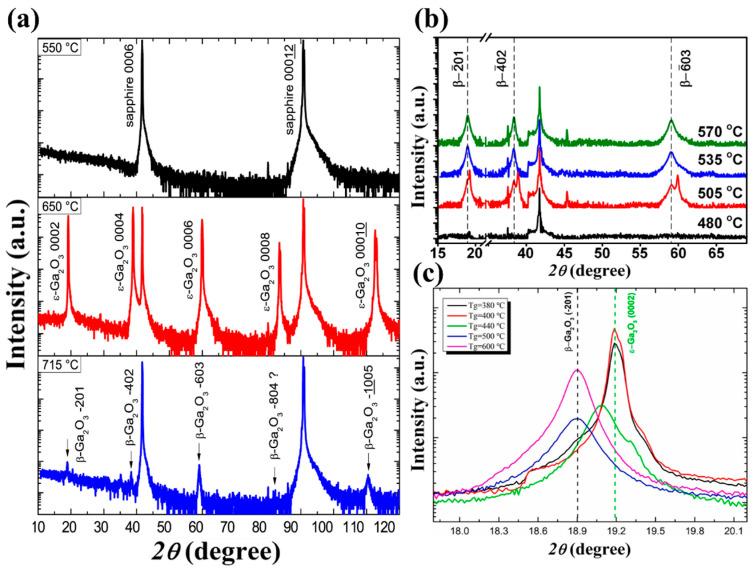
(**a**) XRD spectra of Ga_2_O_3_ samples grown at 550 °C, 650 °C, and 715 °C temperatures on *c*-Al_2_O_3_ [38] (reprinted from [38], with the permission of Elsevier 2016); (**b**) XRD spectra of Ga_2_O_3_ thin films grown at 570 °C, 535 °C, 505 °C, and 480 °C [34] (reprinted from [34], with the permission of Elsevier 2017); (**c**) partially enlarged XRD image of Ga_2_O_3_ films grown under different temperatures [73] (reprinted from [73], with the permission of Elsevier 2021).

The deposition temperature is a crucial factor that directly affects the phase composition and structural composition of the obtained products in CVD systems. Figure 4 shows the XRD spectra of Ga_2_O_3_ films grown at different temperatures by MOCVD. Although the specific conditions of preparation are different in various studies (Table 2 for details), the influence of temperature effect on the film phase and crystallinity remains similar. Figure 4a shows the XRD spectra of the Ga_2_O_3_ grown on *c*-Al_2_O_3_ at different temperatures. At 550 °C, the film shows no diffraction peak of *β* phase or *ε* phase, indicating an amorphous or nanocrystalline quality. When temperature rises to 650 °C, XRD spectra illustrates the high intensity with narrow full width at half maxima diffraction (FWHM) peak of 0.3°, all belong to *ε*-Ga_2_O_3_. This suggests that the film is a pure *ε* phase film, with high epitaxial quality. The diffraction peak of the *ε*-Ga_2_O_3_ (0002) crystal plane was confirmed by comparison [38]. With a further increase in temperature of up to 715 °C, all *ε*-Ga_2_O_3_ peaks disappear, while weak peaks belong to *β* phase show up (the film tends to form a low-quality *β* phase at such a high deposition temperature [38]).

Figure 4b demonstrates similar phase transition trends under varied precursor flow rates and growth pressures. Despite the fact that the specific temperature for forming the *ε* phase varies a lot, the trends of changes in the film phase and quality are similar to those of the previous case. The *ε* phase only stabilizes at a medium temperature. Lower temperatures will lead to a low-crystallinity film, while high temperatures will yield a pure *β* phase Ga_2_O_3_ film [34]. Zhang et al. [73] used oxygen and TEGa as precursors with nitrogen (N_2_) as carrier gas to obtain Ga_2_O_3_ on *c*-Al_2_O_3_. As the temperature changes, the phase composition of the prepared film changes in the same way as before the growth of the film from the pure *ε* phase to the *β* phase with increase in growth temperature (Figure 4c).

**Figure 5:**
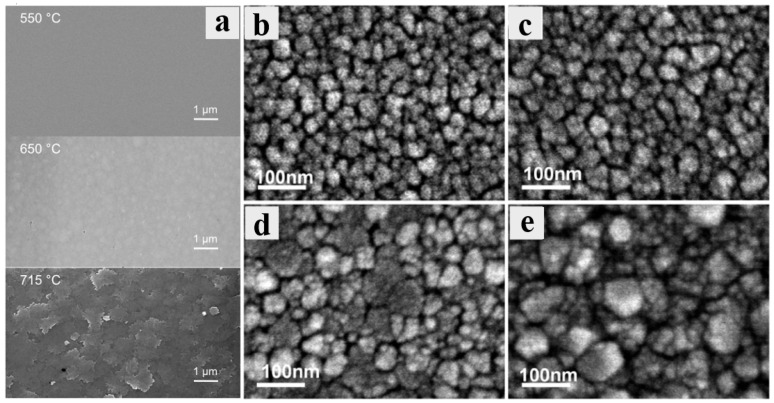
(**a**) SEM images of Ga_2_O_3_ samples grown at 550 °C, 650 °C, and 715 °C on *c*-Al_2_O_3_ [38] (reprinted from [38], with the permission of Elsevier 2016). SEM images of Ga_2_O_3_ thin films grown at (**b**) 570 °C, (**c**) 535 °C, (**d**) 505 °C, and (**e**) 480 °C [34] (reprinted from [34], with the permission of Elsevier 2017).

Figure 5 shows the surface morphologies of films grown at different temperatures. Figure 5a is the SEM images corresponding to the sample of Figure 4a. The XRD spectrum indicates that an amorphous layer is obtained at a low temperature, despite the film being exceptionally smooth. As the temperature rises, the pure *ε* phase is formed, and the SEM images reveal large grains with surface roughness ranging from 1 nm to 4 nm (the closer to the gas inlet, the rougher the surface will be) [38]. However, the surface roughness of the film prepared at 715 °C exhibited a notable increase, accompanied by a considerable grain boundary blurring. As illustrated in Figure 5a, this phenomenon can be attributed to the dissolution of *ε* phase and the emergence of the *β* phase, which exhibits inferior crystalline characteristics. Figure 5b–e show the SEM images of the samples from Figure 4b. When the growth temperature drops, the SEM image exhibits distinct morphological evolution. Figure 5d corresponds to the SEM image at 505 °C, and the XRD results confirm that *ε*-Ga_2_O_3_ grains appear at this temperature. There are two kinds of grains with varied sizes in the SEM image, but they do not appear at higher temperatures. The larger of these grains correspond to the (0001) orientation *ε*-Ga_2_O_3_ [34].

In brief summary, although the *ε* phase is obtained under different deposition parameters, the effect of temperature on the formation of the *ε* phase follows a consistent trend. The optimal growth temperature window for the *ε* phase is lower than that for the *β* phase. The amorphous phase tends to be generated when the temperature is incredibly low. With the growth temperature increases, the *ε* phase will first appear. When the growth temperature continues to a high value, the *β* phase will be generated, suppressing the *ε* phase’s growth and ultimately yielding pure *β*-Ga_2_O_3_. The range of temperature windows suitable for the growth of the *ε* phase is relatively narrow. The optimal growth temperature window for the *ε* phase is determined by the specific preparation process, and the growth temperature window varies significantly with experimental conditions; this also leads to difficulties in determining the temperature that is suitable for the growth of the *ε* phase.

#### 2.1.3. Growth Pressure

**Figure 6:**
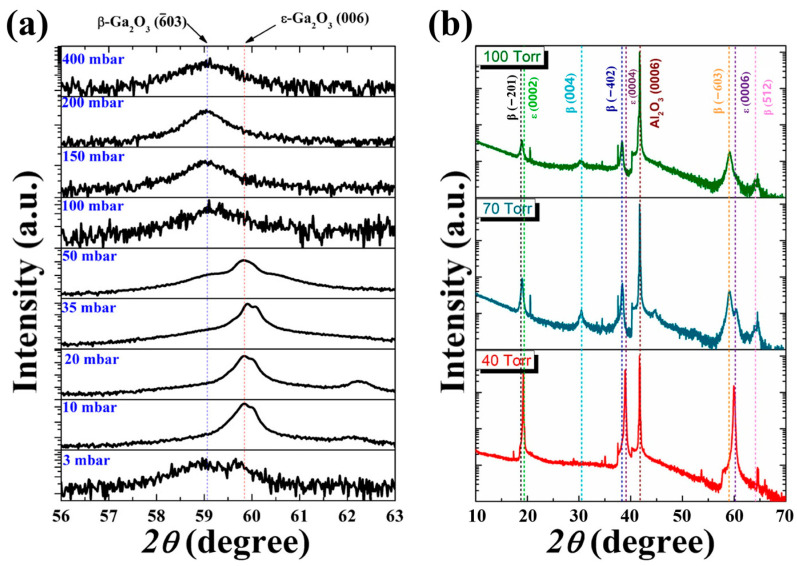
(**a**) XRD spectra of Ga_2_O_3_ films prepared at different pressures from 3 mbar to 400 mbar between 56° and 63° [75] (reprinted from [75], with the permission of ACS Publications 2018); (**b**) XRD spectra of Ga_2_O_3_ films grown under different temperatures [73] (reprinted from [73], with the permission of Elsevier 2021).

In 2013, Playford et al. [74] studied the effects of growth pressure on the phase composition of Ga_2_O_3_ film. In the locally amplified image of high-angle X-ray diffraction (Figure 6a), the diffraction peaks of *β*-Ga_2_O_3_ and *ε*-Ga_2_O_3_ appear simultaneously under the growth condition of very low initial pressure (3 mbar), revealing polycrystalline characteristics. Increasing growth pressure eliminates *β* phase diffraction peaks in high-angle regions, though residual *β* phase signatures persist at low angles. This finding demonstrated the pressure-dependent suppression of *β* phase growth and *ε* phase dominance in the low-pressure range. As the growth pressure further increases, the film should be a pure *ε* phase film. As the growth pressure increases to a higher level (50 mbar), a miscible film in the *β* and *ε* phases can be obtained, and the film ultimately transitions to pure *β*-Ga_2_O_3_ as the growth pressure continues to increase.

The reason for phase formations with growth pressure originates from the presence of heterogeneous nucleation mechanisms and carbon residue (introduced by the precursor). Residual carbon acts as a nucleation site of stable *β*-Ga_2_O_3_ crystal nuclei under low-pressure conditions, which promotes the formation of the *β* phase and promotes the growth of the *ε* phase under suitable pressure conditions. High-pressure homogeneous nucleation preferentially stabilizes thermodynamically favored *β* phase nuclei [75].

In Figure 6b, we can see that Zhang et al. [73] observed that, at low growth pressure, the film is pure *ε* phase; as the growth pressure increases, the composition of the film gradually changes to a mixture of *ε* and *β*. As the pressure continues to rise, the film becomes a pure *β*-phase film eventually. Chen et al. [37] epitaxially grew Ga_2_O_3_ thin films under different growth pressure conditions, and the films obtained at lower growth pressures have better crystal quality (analyzed by XRD spectra). Zhao et al. [65] fabricated Ga_2_O_3_ on *α*-Al_2_O_3_ at different pressures. At a low pressure, the deposited film is a mixed phase of *ε* and *β*, but when the growth pressure is increased to 20 Torr, only *β*-Ga_2_O_3_ related diffraction peaks appear. This also indicated that high-pressure will inhibit the growth of *ε* phase, and the crystal is more inclined to grow into the most stable *β*-Ga_2_O_3_.

**Figure 7:**
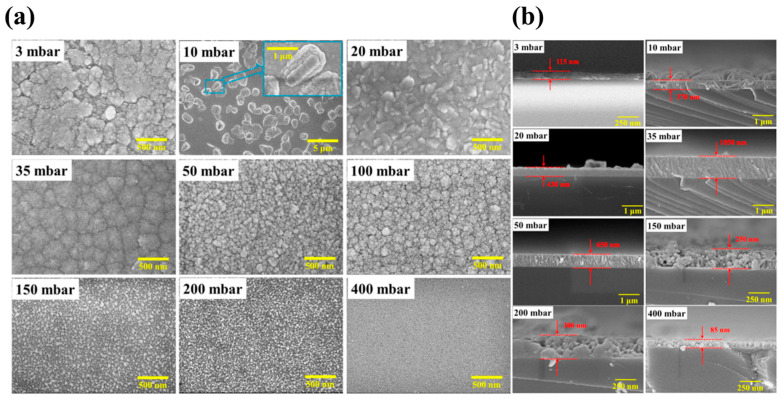
(**a**) Surface morphology and (**b**) the thickness of *ε*-Ga_2_O_3_ films prepared at different pressures from 3 mbar to 400 mbar [75]. Reprinted from [75], with the permission of ACS Publications 2018.

Figure 7 shows the SEM surface and sectional images corresponding to the sample in Figure 6a. Coarse nano- or microscopic structures can be observed at low growth pressures (3–20 mbar). As the pressure gradually increases, nanoparticle coarsening occurs. This is consistent with the analysis of residual carbon which promotes *β*-Ga_2_O_3_ nucleation. At medium growth pressure, the surface is smooth and the grain boundaries are evident, which is due to the combined action of two factors. The first is the lattice mismatch stress and the other is the ability of the growth atoms to migrate to the surface at a lower temperature. XRD spectra confirms that the film is in pure *ε* phase under this condition [75].

Growth pressure not only affects the phase composition of the film, but also greatly influences the growth rate of the film. The growth pressure is usually negatively correlated with the growth rate of the film. On one hand, the increase in growth pressure will shorten the average molecular free path of gas phase particles in the reaction chamber and increase the probability of effective collision between reactants. A large amount of the reactant is consumed which reduces the growth rate. At the same time, the material diffusion coefficient decreases and the growth rate decreases. On the other hand, the precursors during MOCVD processes will participate in two reactions, the parasitic gas-phase reaction and the film growth reaction [76]. With the increase in growth pressure, the parasitic gas-phase reaction was enhanced because of the lowering down of its Gibbs free energy [75,77]. Thus, more reactive precursors will be consumed in the parasitic gas-phase reaction and lead to the inhibition of the film growth rate.

**Figure 8:**
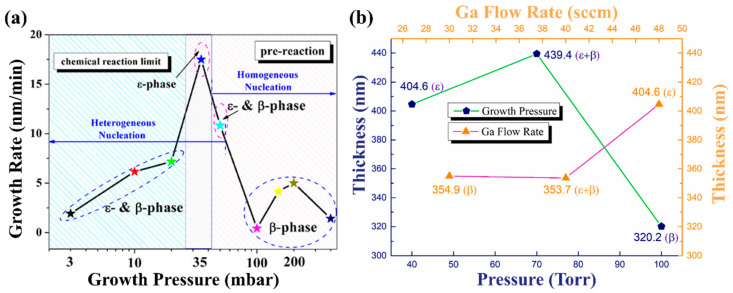
(**a**) Growth rate, crystal phase, and nucleation change of Ga_2_O_3_ with growth pressure [75]; (**b**) thickness of the Ga_2_O_3_ films with different growth pressures [73]. Reprinted from [73], with the permission of Elsevier 2021.

According to the SEM cross-sections images corresponding to different pressures in Figure 7 and Figure 8a, with the increase in growth pressure, the deposition rate has a trend of first rising and then decreasing. When the growth pressure goes up to a value, the deposition rate decreases significantly. This is mainly because high pressure greatly enhances parasitic gas-phase reactions, which lead to large consumption of metal–organic precursors and formation of nanoparticles. High growth pressure will cause uniform nucleated spherical Ga_2_O_3_ nuclei to grow in the gas phase and take advantage of epitaxial nucleation on the substrate surface instead of homogeneous nucleation [75]. The film thickness and deposition rate under different growth pressures also reflect the same phenomenon in Figure 8b. The film thickness increases with the pressure goes up, but excessive growth pressure will eventually lead to a decrease in deposition rate [73].

**Figure 9:**
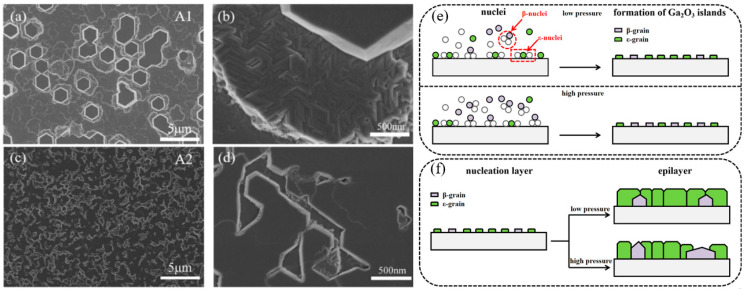
SEM images of *ε*-Ga_2_O_3_, (**a**,**b**) grown at 80 Torr, (**c**,**d**) grown at 60 Torr; schematic diagrams of (**e**) the nucleation stage and (**f**) the epilayer stage [37]. Reprinted from [37], with the permission of Elsevier 2022.

The influence of growth pressure on the growth film discussed above all refers to the influence on the epitaxial stage of the film layer. The SEM image in Figure 9 reflects the influence of different growth pressures on the nucleation stage. In the case of nucleation under lower growth pressure (Figure 9c), the coverage of crystal nuclei in the substrate is better than that under higher growth pressure (Figure 9a), which also indicates that low growth pressure can promote the formation of *ε*-Ga_2_O_3_ in the nucleation stage and inhibit *β*-Ga_2_O_3_. In addition, the growth of the film is transformed from 3D growth mode to 2D, but too low growth pressure will lead to a slowing down of the growth rate [37].

In summary, growth pressure plays a decisive role in the formation of *ε*-Ga_2_O_3_ in MOCVD system. Lower growth pressure is conducive to promoting the formation of *ε*-Ga_2_O_3_ and inhibiting the formation of *β*-Ga_2_O_3_. However, too low growth pressure will lead to carbon residue via MOCVD technique, and carbon acts as the stable nucleation site of *β*-Ga_2_O_3_ to promote the formation of the *β* phase, resulting in the inability to obtain the pure *ε* phase. In the film nucleation stage, lower growth pressure can also inhibit the nucleation of *β*-Ga_2_O_3_, promote the nucleation of *ε*-Ga_2_O_3_, and change the growth mode of the film from 3D mode to 2D mode.

#### 2.1.4. Precursor Ratio

**Figure 10:**
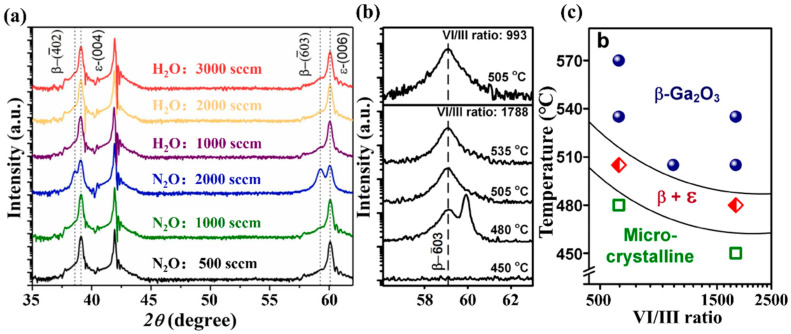
(**a**) XRD spectra of Ga_2_O_3_ films grown at different VI/III ratio environment [78] (reprinted from [78], with the permission of Elsevier 2021). (**b**) XRD spectra of Ga_2_O_3_ films grown at various temperatures with higher VI/III ratios [34]; (**c**) phase diagram of Ga_2_O_3_ films grown at constant TEGa flow rate of 67.4 μmol/min and various VI/III ratio environment [34] (reprinted from [34], with the permission of Elsevier 2017).

Precursor ratio (denoted as VI/III ratio) refers to the molar flow ratio of group VI atom (O) and group III atom (Ga) during MOCVD growth. Fei et al. [78] have reported the growth of Ga_2_O_3_ by MOCVD via change the type and ratio of precursor. Figure 10a shows the XRD spectrum of *ε*-Ga_2_O_3_ prepared under different oxygen sources and VI/III ratios conditions. As H_2_O as the oxygen source, increasing gas flow rate did not significantly change the film phase, which can be owning to the VI/III ratio remaining within the growth window of *ε*-Ga_2_O_3_ [38]. However, the sample changes the growth precursor from H_2_O 2000 sccm to N_2_O. A value of 2000 sccm indicates the mixture phase of *β* and *ε* in the XRD spectra, indicating that phase composition depends on oxygen source type; in general, *ε* is stable under low VI/III ratio preparation conditions. Zhou et al. [34] studied the coupling effects of temperature and precursor ratio on the growth phase composition of films. *ε*-Ga_2_O_3_ was absent under growth conditions with a high VI/III ratio, but emerged when temperature was reduced at this VI/III ratio, as shown in Figure 10b. Figure 10c also presents the phase diagram under the combined action of temperature and VI/III ratio, which is used to guide the synthesis of films composed of specific phases.

Jiang et al. [79] observed that modulation of the precursor ratio in the preparation of Ga_2_O_3_ by MOCVD leads to a simultaneous change in the growth rate of the film, and that the growth rate also plays an important role in determining the formation of the film phase. However, this effect can often be attributed to the change in precursor rather than the growth rate. In this study, the growth rate increases with the increase in Ga ions flow rate. As seen from the X-ray diffraction spectra, *β*-Ga_2_O_3_ appears with the lowest growth rate (as well as the largest VI/III ratio). However, with the increase in growth rate (i.e., VI/III ratio decreases), the diffraction of *β*-Ga_2_O_3_ disappears, indicating the presence of pure *ε*-Ga_2_O_3_; the intensity of the peak belonging to *ε*-Ga_2_O_3_ keeps increasing with decreases in the VI/III ratio. This phenomenon demonstrates that a reduction in the VI/III ratio can inhibit the formation of the *β* phase and promote the growth of the *ε* phase. Synthesizing the above, a lower ratio and a Ga-rich environment will contribute to the generation of *ε*-Ga_2_O_3_.

#### 2.1.5. Special Deposition Techniques

##### Precursors Type

**Figure 11:**
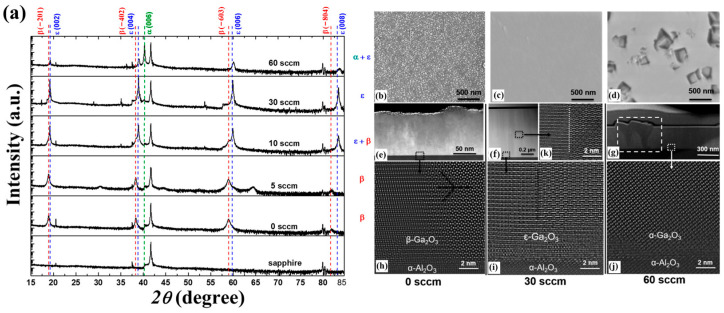
(**a**) XRD spectra of Ga_2_O_3_ films grown under different flow rates of HCl; SEM images of the Ga_2_O_3_ films grown under (**b**) 0 sccm, (**c**) 30 sccm, and (**d**) 60 sccm of HCl; HAADF STEM imaging of FIB cross-sectional samples grown under HCl flow rates of (**e**) 0 sccm, (**f**) 30 sccm, and (**g**) 60 sccm (the dashed box region indicates the hybrid growth of the film (*α*- and *ε*-Ga_2_O_3)_); (**h**–**j**) high-magnification STEM images (Gaussian high-pass filtered image) collected at the interface of the three samples, respectively; (**k**) detail at the domain boundary along the film [45]. Reprinted from [45], with the permission of ACS Publications 2018.

As the raw material of the reaction, the precursors critically govern the formation of the product. Sun et al. [45] regulated the phase composition and structure of Ga_2_O_3_ films by introducing HCl into the precursors. XRD spectra of films grown under different HCl fluxes are shown in Figure 11a. With the increase in HCl flux, the film illustrated the transformation process from pure *β* phase to a mixture phase with *β* and *ε*, then to pure *ε* phase, and eventually to a mixed phase of *ε* and α. It is worth mentioning that the Cl^−^ content in the epitaxy layer did not increase, despite increasing the HCl flux in the precursor. This observation indicates that HCl does not participate in the reaction but acts as a catalyst to promote the reaction.

**Figure 12:**
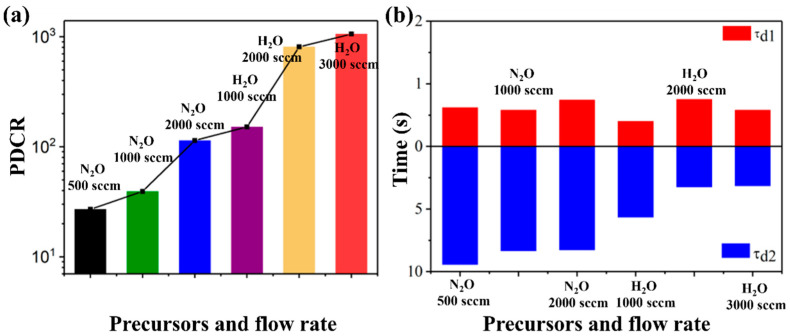
(**a**) Photo-to-dark current ratio and (**b**) τ_d1_ and τ_d2_ of Ga_2_O_3_ photodetectors grown with different oxygen source under a bias of 15 V [78]. Reprinted from [78], with the permission of Elsevier 2022.

Fei et al. [78] compared the effects of different oxygen sources (H_2_O/N_2_O) on the growth of *ε*-Ga_2_O_3_ in MOCVD. Meanwhile, a photodetector was fabricated based on the prepared *ε*-Ga_2_O_3_, and the influence of different oxygen sources on the photodetector was studied. The study shows that compared with N_2_O, the *ε*-Ga_2_O_3_ thin films grown with H_2_O as oxygen source have higher crystallinity, fewer defects, a fast response speed with a rise time of 0.55 s, and a decay time of 0.58 s/3.14 s; the highest photo-to-dark current ratio (PDCR) of the photodetector reaches to 1 × 10^3^ (shown in Figure 12), owing to the fact that H_2_O, as an oxygen source, is more effective for filling oxygen vacancies in *ε*-Ga_2_O_3_, which reduces defect density and improves performance.

##### Growth Pattern

**Figure 13:**
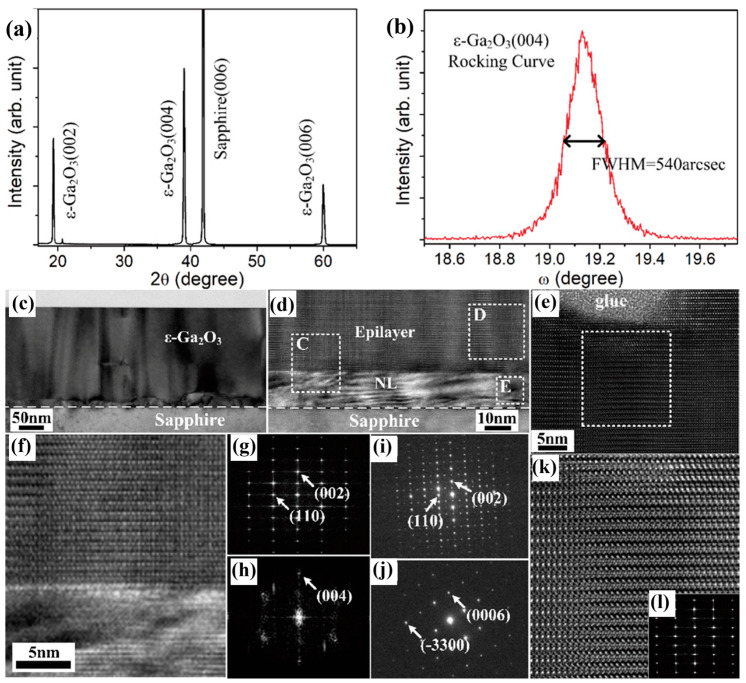
(**a**) XRD patterns of the *ε*-Ga_2_O_3_ thin film; (**b**) XRD rocking curve of the *ε*-Ga_2_O_3_ (004) plane; (**c**) image of the whole film; the substrate/film interface is indicated by the dashed line; (**d**) image of a region near the film/substrate interface; (**e**) magnified image of region C indicated in (**d**); FFTs of the regions (**f**) D and (**g**) E indicated in (**d**); selected-area electron diffraction (SAED) patterns of the (**h**) *ε*-Ga_2_O_3_ epilayer and (**i**) sapphire substrate; (**j**) image of the bottom of a pinhole; (**k**) FFT and (**l**) inverse FFT of the region indicated in (**j**) [80]. Reprinted from [80], with the permission of IOP Science 2018.

Multilayer growth modes typically result in high-density defects and surface roughness, which have great adverse effects on the application of electronic devices. In order to improve the crystal quality of the grown *ε*-Ga_2_O_3_, Chen et al. [80] used a two-step growth method to first grow the nucleation layer (NL) under certain conditions, and then changed the conditions to grow the epitaxial layer. This two-step growth mode successfully shifts the epitaxial layer from multilayer growth mode to layer-by-layer growth mode, which can effectively promote the growth quality of epitaxial layer. Figure 13a,b characterized the crystal quality of *ε*-Ga_2_O_3_ with a maximum FWHM of 540 arcsec (0.15°). In the TEM cross-section images (Figure 13), three layers of substrate, a nucleating layer, and *ε*-Ga_2_O_3_, as well as two interfaces of substrate/NL and NL/epitaxial layer, can be clearly identified. The NL grew at the first stage to relax lattice strain between epitaxial layer and substrate, providing *ε*-Ga_2_O_3_ with an epitaxial nucleation condition. The layer-by-layer film growth is then achieved by increasing the growth temperature, and the newly formed *ε*-Ga_2_O_3_ grains can be fully expanded laterally. The layer-by-layer growth mode leads to a reduction in defects while increasing in crystallinity.

### 2.2. Physical Vapor Deposition Method

Compared with the CVD methods, the research of *ε*-Ga_2_O_3_ films synthesized by the PVD methods is much less reported. As early as 2002, Masahiro Orita et al. [81] reported that the *ε* phase will show up in a *β*-Ga_2_O_3_ thin film with Sn doping using the PLD technique. In that period, the importance of the *ε* phase was not seriously considered. And efforts have been focused on obtaining pure *β* phase films from preventing the *ε* phase. However, Sn doping has currently become the most popular approach for obtaining *ε*-Ga_2_O_3_ by PVD. It is worth mentioning that the PVD technique is promising for obtaining pure *ε* phase, as it can reduce the introduction of unintentional doping impurities during the deposition process and has almost no reaction by-products. Precise control of the stoichiometric ratio can be achieved by PVD techniques such as PLD [75]. PLD and MBE are the most commonly used PVD techniques to synthesize *ε*-Ga_2_O_3_ films. Because of the different thermodynamic and kinetic principles of PVD and CVD, the deposition parameters and corresponding effects on the film structures are quite different. This section will focus on reviewing the effects of key parameters in PVD techniques on Ga_2_O_3_ film’s growth.

#### 2.2.1. Temperature, Oxygen Pressure, and Deposition Energy

As with chemical vapor deposition, the deposition temperature usually determines the phase composition of the obtained film. In 2007, Yoshioka H. et al. [82] conducted first-principle calculations for five Ga_2_O_3_ polymorphs, which predicted that the difference in free energy between the *β* phase and the *ε* phase becomes smaller through increasing the temperature to obtain pure *ε*-Ga_2_O_3_.

**Figure 14:**
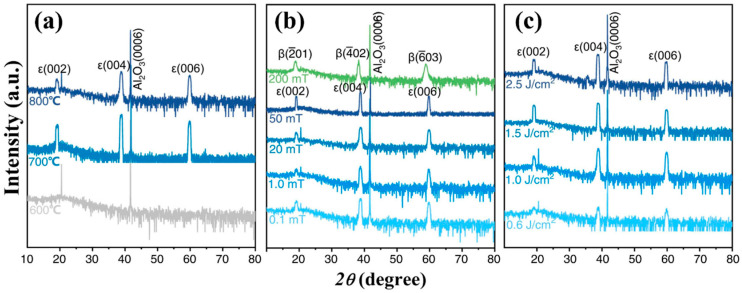
X-ray diffraction patterns of 1 mol % Sn-doped Ga_2_O_3_ films deposited on *c*-Al_2_O_3_ substrates (**a**) at temperatures of 600–800 °C, (**b**) at oxygen pressures of 0.1–200 mTorr, and (**c**) at laser fluence of 0.6–2.5 J/cm^2^ [62]. Reprinted from [62], with the permission of Elsevier 2022.

Zhang et al. [62] studied the effects of temperature, oxygen pressure, and deposition energy on Sn doping-assisted growth of *ε*-Ga_2_O_3_. Figure 14a reflects the influence of different substrate temperatures on the growth phase composition of Ga_2_O_3_ films. XRD spectra show that a high crystal quality of *ε*-Ga_2_O_3_ grows at both 700 °C and 800 °C, but amorphous or nanocrystalline forms appear at 600 °C. However, this does not mean that a higher substrate temperature is conducive to the generation of the *ε* phase, because films grown at 700 °C show a significant diffraction peak intensity with a narrower FWHM than that obtained at 800 °C. These phenomena indicate that there is a suitable temperature range for the generation of the *ε* phase, similar to the mode of the temperature action in CVD to prepare *ε*-Ga_2_O_3_, as described in Section 2.1.2.

Figure 14b shows the influence of oxygen pressure on the preparation of *ε*-Ga_2_O_3_ via Sn doped by PLD. In XRD spectra, *ε*-Ga_2_O_3_ correlating diffraction peaks appear when the oxygen pressure is below 50 mTorr. With the increase in oxygen pressure, the diffraction peak intensity increases and the FWHM slightly decreases, which indicates that the quality of the *ε* phase increases with the increase in oxygen pressure. However, when the oxygen pressure reaches 200 mTorr, the film is no longer *ε*-Ga_2_O_3_ but completely *β*-Ga_2_O_3_. Moreover, the FWHM of films at a high angle is wider than that of the *ε*-Ga_2_O_3_ diffraction peak prepared at a low oxygen pressure, which indicates a poor crystallinity in the *β* phase.

Gao et al. [60] prepared tin-assisted Zr-doped n-type *ε*-Ga_2_O_3_ thin films by adjusting growth parameters of PLD (oxygen pressure and deposition pulsed number). The study illustrated that, under high oxygen pressure (≥0.06 mbar), the phase composition of the film changed from pure *ε* phase to a mixed phase of *β* and *ε*. With the increase in oxygen pressure, the grain size increases while the FWHM of *ε*-Ga_2_O_3_ decreases. The same effect of oxygen pressure also appears in the preparation of *ε*-Ga_2_O_3_ by MBE. Kracht et al. [64] adjusted the deposition environment by changing the Ga flux under the condition of fixed Sn flux and oxygen flux, and the results showed that the formation of *β* phase would be promoted with an oxygen-rich environment, while the formation of *ε* phase would be promoted under the condition of Ga enrichment. Ardenghi et al. [63] also indicated that the films obtained at higher Ga^+^ fluxes are pure *ε* phase, while lower cationic fluxes will cause the films to be a miscible phase.

The oxygen pressure in PVD technique is similar to the VI/III ratio in MOCVD. From the previous discussion on VI/III ratio in CVD, we can draw a general conclusion that high VI/III ratio will inhibit the growth of *ε*-Ga_2_O_3_. A high VI/III ratio means the proportion of oxygen element in the growth process increases, which is similar to the increase in oxygen pressure in PLD. Therefore, we can draw a tentative conclusion that sufficient oxygen sources can promote the formation of *β*-Ga_2_O_3_ and inhibit the formation of *ε*-Ga_2_O_3_ in both CVD and PVD. Although the effect of oxygen pressure on the formation of Ga_2_O_3_ phase composition can be simply analyzed by XRD, no research has been able to explain the mechanism of oxygen pressure on the change of film phase composition. There are some explanations for this: that the low oxygen pressure reduced the free energy of *ε*-Ga_2_O_3_; that the low oxygen pressure causes the film to form Ga_2_O rather than Ga_2_O_3_ crystal nuclei directly during the growth process; that SnO and SnO_2_ can suppress the desorption of Ga_2_O and a Ga-rich environment remains, thus promoting the generation of the *ε* phase, meaning that more Ga_2_O generation will promote the generation of *ε*-Ga_2_O_3_. The following third section explains the possible mechanisms of *ε*-Ga_2_O_3_ growth.

It is also important to consider the energy in deposition. Generally, the particles deposited on the film have higher kinetic energy with higher energy which provides enough energy for nucleation and diffusion. In PLD, deposition energy is usually changed by changing the laser fluence. Figure 14c explores the phase composition of Ga_2_O_3_ film grown under different deposition energies. In the XRD results, the crystal quality of the *ε* phase is significantly improved with the increase in laser fluence, especially when the laser energy is low, and the effect on the crystal quality of the film is not obvious when the laser energy is high. By comprehensive analysis, substrate temperature and oxygen pressure are the key factors affecting the preparation of *ε*-Ga_2_O_3_ by PLD. Low oxygen pressure and appropriate substrate temperature are the necessary growth conditions for *ε*-Ga_2_O_3_; meanwhile, the laser energy window required for growth of *ε*-Ga_2_O_3_ is wide, and laser fluence affects the crystal quality and has less influence on the phase composition than oxygen pressure and substrate temperature.

#### 2.2.2. Doping

**Figure 15:**
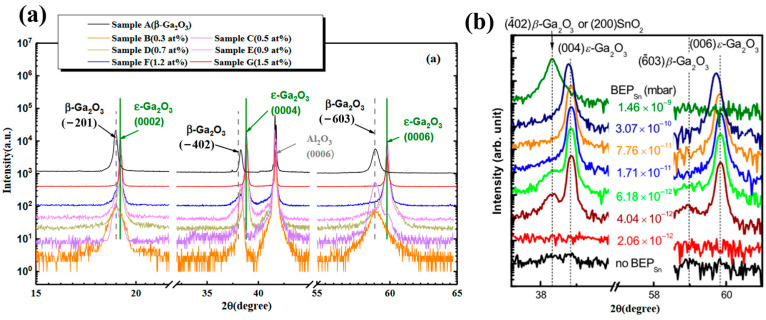
(**a**) HRXRD diffraction curves of Sn assisted growth of *ε*-Ga_2_O_3_ and undoped *β*-Ga_2_O_3_ samples on sapphire [53] (reprinted from [53], with the permission of Optica Publishing Group 2018); (**b**) *ω*-2*θ* scans of the samples grown at a BEP_Ga_ of 1.77 × 10^−7^ mbar and at different BEP_Sn_ values [64] (reprinted from [64], with the permission of APS 2017).

Doping is a necessary condition in current studies of *ε*-Ga_2_O_3_ obtained via PVD methods. At present, there have been no reports of *ε* phase synthesized by PVD techniques without doping. Almost all the existing reports used Sn or In doping. Cai et al. [53] used PLD to prepare *ε*-Ga_2_O_3_ and studied the effect of Sn doping content on the phase composition of the films. Figure 15a shows the XRD spectra, indicated that the samples only with Sn-doped ≥ 0.7at% form *ε*-Ga_2_O_3_, and their crystal quality improves significantly with the increasing Sn content. Similar phenomenon also appears in *ε*-Ga_2_O_3_ deposition by molecular beam epitaxy. Figure 15b shows the XRD results of Ga_2_O_3_ prepared by changing the BEP_sn_ value in molecular beam epitaxy. With the increase in BEP_sn_ value, the film changes from a mixed phase of *β*-Ga_2_O_3_ and *ε*-Ga_2_O_3_ to pure *ε*-Ga_2_O_3_, but when BEP_sn_ continues to increase, there is only one low-angle diffraction peak belonging to SnO_2_, and the Ga_2_O_3_ becomes amorphous under this condition. This is due to excess formation of SnO_2_, which impairs the crystallization of *ε*-Ga_2_O_3_. Ardenghi et al. [63] confirmed that under suitable deposition conditions, indium can also be an effective *ε* phase stabilizing doping element. So far, the existence of Sn and In in *ε*-Ga_2_O_3_ is still unclear.

Our team used PLD to deposit Ga_2_O_3_ films with varying Sn contents to investigate the influence of Sn content on the formed phases of Ga_2_O_3_ films. When the proportion of Sn in the total metal atoms is less than 0.2%, the film is in the pure *β* phase. Further increase in Sn concentration leads to a mixture of *β* and *ε* phases in the film. As the Sn content in the film increases to 0.5 at%, a pure *ε* phase film can be obtained. Further increasing the Sn content to 1 at% does not affect the phase or crystallinity of the film. And no SnO or SnO_2_ precipitates are observed. Our work also shows that Sn acts as an n-type doping element only in the *β* phase but acts as an element to stabilize the phase in the *ε* phase. When the film is in pure *β* phase or is mixed with more *β* phase, increases in Sn content will lead to an increase in the film carrier density and thus in conductivity. Once the *ε* phase increases to a certain ratio in the film, Sn is segregated in the *ε* phase with much less Sn doped in the *β* phase, leading to a significant drop in film conductivity.

#### 2.2.3. Substrate

**Figure 16:**
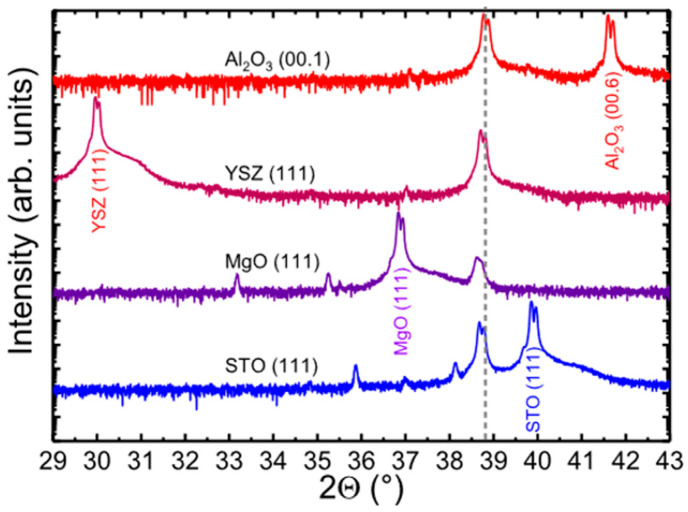
XRD spectra around the *ε*-Ga_2_O_3_ (004) reflection for thin films deposited on *c*-Al_2_O_3_, MgO (111), SrTiO_3_ (111), and yttria-stabilized ZrO_2_ (111) substrates [54]. Reprinted from [54], with the permission of AIP Publishing 2019.

Different from the preparation of *ε*-Ga_2_O_3_ by CVD technique, the reports related to the substrate effects on the synthesis of *ε*-Ga_2_O_3_ by PVD methods are far fewer. *c*-Al_2_O_3_ is the most commonly used substrate for the PVD growth of *ε*-Ga_2_O_3_. M. Kneiss et al. [54] have studied the growth of *ε*-Ga_2_O_3_ films on *c*-Al_2_O_3_, MgO (111), SrTiO_3_ (111), and YSZ (111) substrates via PLD, and XRD spectra of films grown on different substrates are shown in Figure 16. The *ε*-Ga_2_O_3_ was successfully obtained on all kinds of substrates with Sn doping. The in-plane and out-of-plane lattice-matching relationships between *ε* phase and substrates are the same as those reported in CVD [31,38,49,65]. Notably, the *ε* phase shows a much smaller lattice mismatch between MgO (111) substrates compared with *c*-Al_2_O_3_ and YSZ (111) substrates, but it exhibits the lowest crystal quality according to the XRD spectra. This implies that the lattice-matching relationship plays a less important role for the growth of *ε*-Ga_2_O_3_ via PVD techniques. Unlike the cases of CVD methods, the *ε* phase is mainly stabilized by dopants in PVD cases, and its strain status can be influenced by the selection of substrates or buffer layers.

## 3. Forming Mechanisms of *ε*-Ga_2_O_3_

There have been many reports about the successful depositions of *ε*-Ga_2_O_3_ using CVD or PVD. The effects of various growth parameters on the film phase composition have been systematically explored. However, there have been no conclusive opinions about the formation mechanisms of *ε*-Ga_2_O_3_. At present, several conjectures have been proposed, which can be divided into two categories according to the differences between CVD and PVD techniques. One hypothesis is that 2D growth [37,83], also known as layer-by-layer growth [80], promotes the formation and stability of *ε*-Ga_2_O_3_. This theory has been applied to explain the film formation mechanism of *ε* phase prepared by CVD. Another mechanism is related to the specific lattice occupation of Sn dopants, which has been used to explain the formation of the *ε* phase using PVD techniques.

**Figure 17:**
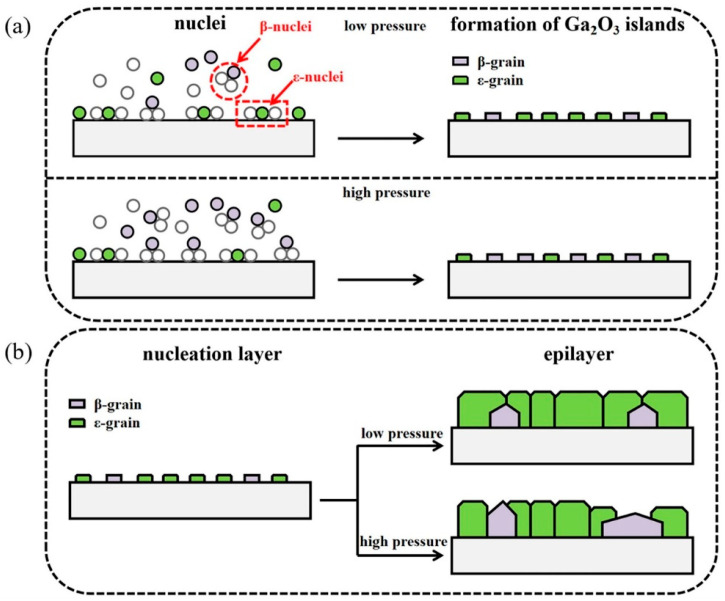
Schematic diagrams of Ga_2_O_3_ structure revolution: (**a**) the nucleation stage and (**b**) the epilayer stage [37]. Reprinted from [37], with the permission of Elsevier 2022.

When using CVD techniques to grow ε-Ga_2_O_3_ films, a layer-by-layer growth mode is beneficial for inhibiting the longitudinal growth of the crystal nucleus and promoting transverse growth. The possibility of the nucleation of a new atomic layer on the surface can be reduced by diminishing growth pressure. This means that, when an *ε*-Ga_2_O_3_ crystal nucleates on the substrate, the longitudinal growth of grain is suppressed; meanwhile, the generated *ε*-Ga_2_O_3_ nuclei could have ample opportunity to expand and polymerize along the lateral path because of the lower growth rate. Figure 17 gives a schematic drawing of this growth mechanism. In the initial nucleation stage of Ga_2_O_3_ (Figure 17a), the crystal nuclei of *ε*-Ga_2_O_3_ and *β*-Ga_2_O_3_ are both generated on the substrate, and the low growth pressure contributes to the nucleation of *ε*-Ga_2_O_3_. Low pressure, promoting *ε*-Ga_2_O_3_ nucleation, provides a large number of initial positions for the subsequent growth of *ε*-Ga_2_O_3_. When the nucleation stage is over and the crystal nuclei begins to grow, the lower pressure can inhibit the longitudinal growth of the grain. Therefore, the initial grains tend to laterally expand and integrate first and then grow longitudinally. Owing to the low initial pressure, the substrate surface can be considered as an almost completely nucleated layer of *ε*-Ga_2_O_3_. Thus, the subsequent growth process can be considered as a homo-epitaxy on the *ε*-Ga_2_O_3_ layer. When the growth pressure is high, the longitudinal growth rate of the nuclei is intensified, and the nucleation of *β*-Ga_2_O_3_, which is the most thermodynamically stable phase, is facilitated. The rapid longitudinal growth of *β*-Ga_2_O_3_ also hinders the transverse expansion of the *ε*-Ga_2_O_3_ nuclei, which ultimately results in the formation of mixture phase of *β* and *ε*. In this case, excessive longitudinal growth rate (3D growth mode) will lead to a strain relaxation in the film so that the epitaxial layer will be dominated by the most thermodynamically stable *β* phase. In contrast, layer-by-layer growth mode will allow each layer of the Ga_2_O_3_ to get sufficient strain control from the bottom layer and finally lead to an epitaxial growth of *ε* phase.

Despite the successful explanation of *ε*-Ga_2_O_3_ growth after nucleation, 2D growth cannot be called upon to clarify the nucleation mechanism of *ε*-Ga_2_O_3_. Some researchers attributed the nucleation mechanism of *ε*-Ga_2_O_3_ to the substrate. For example, *ε*-Ga_2_O_3_ can nucleate on AlN substrates easier than *β*-Ga_2_O_3_ because both AlN and *ε*-Ga_2_O_3_ have threefold symmetry. This nucleation mode can be considered as the result of strain tuning [22]. Some researchers connected the nucleation mechanism of *ε*-Ga_2_O_3_ with the vapor environments during CVD processes. Sun et al. [45] used MOCVD to achieve the *α*, *β*, and *ε* phases in Ga_2_O_3_ films only by changing the flow rate of HCl. The DFT calculation showed that the inflow of HCl changed the free energy of the three phases. Specific flow rate of HCl could make one of the three phases to be the most stable phase. When there was no HCl in the chamber, the Ga_2_O_3_ was pure *β*-crystalline. As a small amount of HCl was introduced (5 sccm), the growth rate of Ga_2_O_3_ was enhanced. The growth rate of Ga_2_O_3_ peaked when 30 sccm of HCl was passed in, while the film was completely transformed into a pure *ε*-Ga_2_O_3_. Subsequently, the growth rate of Ga_2_O_3_ then decreases with the increase in HCl flux, and the *α*- Ga_2_O_3_ is gradually revealed and dominates with the increase in HCl flux. Although the flow rate of HCl is different, the Cl^−^ content in the film is almost the same. Therefore, HCl mainly plays a catalytic role for *ε* phase nucleation in the growth process of the film.

Interestingly, some researchers reported phenomena contrary to the above mechanism. For example, Jiang et al. [79] reported that low temperature and high growth rate contribute to the formation of *ε*-Ga_2_O_3_ on *c*-Al_2_O_3_ via MOCVD. These opposite results indicate that the mechanisms of the *ε* phase formation are still far from fully understood.

**Figure 18:**
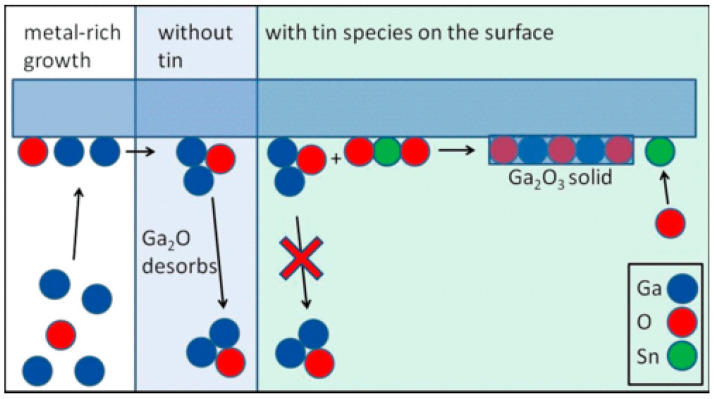
Schematic diagrams for tin-assisted Ga_2_O_3_ growth in metal-rich conditions [64]. Reprinted from [64], with the permission of APS 2017.

In the case of PVD techniques, substrate effects are not as obvious as they are in the CVD case. Compared to the strain tuning from substrates, doping plays a dominant role. Taking Sn as an example, it is now believed that the mechanism of Sn doping can be considered in two aspects. On one hand, Sn can stabilize the growth of Ga_2_O_3_ with a lower VI/III ratio. As illustrated in Figure 18, the oxide of Sn (SnO, SnO_2_) can react with Ga_2_O to transform into Ga_2_O_3_ with a negative ΔG [64,84]. Therefore, the presence of Sn can reduce the desorption of Ga_2_O and maintain the film with ion-rich Ga. As discussed previously, the Ga-rich environment can provide an easier formation of *ε*-Ga_2_O_3_.

On the other hand, Sn^4+^, whose valence state is quadrivalent, accounts for more than 90% of the total Sn atoms in *ε*-Ga_2_O_3_ via XPS analysis. However, XRD spectra have never shown the correlated peaks of SnO_2_ with *ε*-Ga_2_O_3_ simultaneously in the current reports. This implies that Sn atoms enter into the lattice as a substitutional or interstitial impurities [53]. SnO_2_ has a rutile structure, with the Sn and O atoms in an octahedral coordination. In the comparison between the *β* phase and the *ε* phase, it is found that about 75% of Ga atoms in *ε*-Ga_2_O_3_ are on octahedral coordination, while only about 50% of Ga atoms in *β*-Ga_2_O_3_ are on octahedral coordination. The incorporation of Sn can effectively occupy the site of the octahedral lattice and stabilize the octahedral lattice [85]. At the same time, owing to the longer bond length in SnO_2_ than that in the tetrahedral position in Ga_2_O_3_, Sn tends to occupy the octahedral lattice position rather than the tetrahedral lattice position to form *ε*-Ga_2_O_3_ [64,85]. Some researchers also conducted Sn doping studies via Mist-CVD. It was found that the compressive strain induced by Sn could delay strain relaxation and inhibit the transformation of *ε*-Ga_2_O_3_ into more stable *β*-Ga_2_O_3_ [47]. This mechanism can clearly explain the effect of Sn incorporation on the formation of *ε*-Ga_2_O_3_.

Based on the above mechanism, elements with similar characteristics as Sn, such as Si or Ge, should be able to stabilize the *ε* phase of Ga_2_O_3_. However, single doping of Si or Ge has never been proved to be able to get pure *ε* phase. In contrast, our work found that, in the case of co-doping with Sn (fixed as 1at%) and Si, the film changed from pure *ε* phase to *ε*/*β* mixture when Si content increased to more than 0.2%. In addition, some other elements, such as In, were also used to stabilize the *ε* phase. They show quite different characteristics compared with Sn. Therefore, similar to the CVD case, the formation mechanisms of *ε*-Ga_2_O_3_ in PVD case also need further studies.

According to the above discussion, the fundamentals of *ε*-Ga_2_O_3_ growth is to lower down the formation energy of *ε* phase. In the case of CVD, appropriate nucleation conditions of *ε*-Ga_2_O_3_ are established with suitable substrate, temperature, vapor composition and pressure. After nucleation, the layer-by-layer homoepitaxial growth of *ε*-Ga_2_O_3_ will be continued in the same deposition conditions. Therefore, slower growth rate and suitable growth parameters are important in the CVD processes. In the case of PVD, dopants occupy certain positions inside Ga_2_O_3_ lattices, lower down the formation energy of *ε* phase via local bonding. Doping is dominant in both nucleation stage and film growth stage. Therefore, the growth window is wider, and the selection of substrates is more diverse in the PVD processes.

## 4. Deep-Ultraviolet Photodetection Performance of *ε*-Ga_2_O_3_

In the field of photoelectric detection, solar-blind photodetectors (SBPDs) are an indispensable part of spectral detection. The solar-blind region refers to the radiation band ranges from 200 nm to 280 nm, which almost does not exist in the near-earth atmosphere due to the strong absorption of the ozone layer in the atmosphere. Therefore, the detection of this band can greatly reduce the difficulty of external interference and signal processing. SBPDs can play an important role in many key application scenarios, such as fire early warning, missile tracking, and ozone hole monitoring [86]. As a new type of ultra-wide bandgap (~4.9 eV) semiconductor, Ga_2_O_3_ is promising for the deep-ultraviolet photodetectors owing to its environmental stability, high performance, and variety of preparation methods [87,88,89].

Compared with the p-n junction photodetectors for self-bias operation and Schottky junction photodetectors for avalanche multiplication, metal–semiconductor–metal (MSM)-type photodetectors have simpler structure and easier fabrication processes. Therefore, most of the reported *ε*-Ga_2_O_3_ photodetectors are of the MSM type. Table 5 summarized most of the previous research work with the performance of *ε*-Ga_2_O_3_ photodetectors. Similar to the β-Ga_2_O_3_ reports, the photodetection properties of *ε*-Ga_2_O_3_ vary a lot in different studies. The responsivity was reported from hundreds of mA/W to more than 10^4^ A/W. The photo-to-dark current ratio changed from 10^3^ to 10^8^ level. Detectivity and responding time also showed differences in several magnitudes.

The properties of *ε*-Ga_2_O_3_-based photodetectors are heavily affected by many kinds of defects, including grain boundaries, doping induced lattice distortions, and oxygen vacancies, etc. Most defects play negative roles in relation to these properties because of the introduction of trapping energy levels and impedance to carrier migration. The trapping energy levels can lead to the recombination of the photogenerated carriers, while the slow migration of carriers through the defects can delay the response of the photodetectors. In this section, the effects of several common kinds of defects are discussed, the efforts for further improvements of *ε*-Ga_2_O_3_-based photodetectors are reviewed, and the special environmental applications of the photodetectors are summarized.

### 4.1. Crystallinity Effect

The crystal quality of *ε*-Ga_2_O_3_ is sensitive to the substrates according to the previous discussion. Substrates with smaller lattice mismatches with *ε*-Ga_2_O_3_ tend to grow films with higher crystallinity and end up with excellent properties. However, the high cost of some substrates with optima crystal match makes it difficult to commercialize them. In order to expand the applications of *ε*-Ga_2_O_3_, researchers prefer to use low-cost substrates such as Si, which are highly commercialized and easily stripped from the substrate, which also poses challenges for high-quality growth of *ε*-Ga_2_O_3_ on non-optimal lattice mismatch substrates.

**Figure 19:**
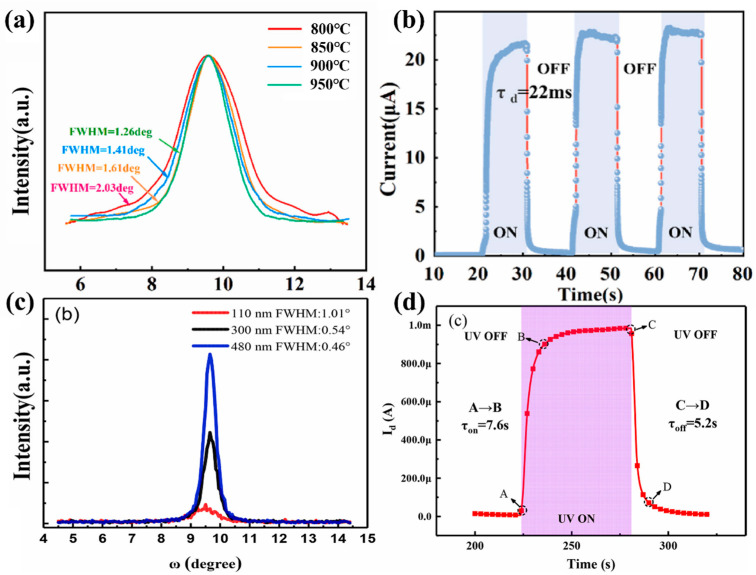
(**a**) XRD rocking of ε-Ga_2_O_3_ (002) epitaxially grown on Mo under different annealing temperatures; (**b**) photoresponse of the ε-Ga_2_O_3_ solar-blind UV photoelectric detector grown on annealed Mo buffer layer [94] (reprinted from [94], with the permission of Elsevier 2023); (**c**) the rocking curve of *ε*-Ga_2_O_3_ films with different thickness [35]; (**d**) transient response of the photodetector to 254 nm illumination at the bias of 5 V correspond to 480 nm in (**c**) [35] (reprinted from [35], with the permission of Elsevier 2021).

With the purpose of alleviating the lattice mismatch between epitaxial film and substrates, it is common to introduce a buffer layer on the substrates. Hu et al. [94] proposed the introduction of an annealed molybdenum (Mo) buffer layer to alleviate the lattice mismatch between Si (100)/AlN and *ε*-Ga_2_O_3_. The XRD rocking curves illustrated FWHM of *ε*-Ga_2_O_3_ (002) plane decrease with Mo buffer layer insertion. They also illustrated that the high-temperature-annealed interlayer is more favorable for the growth of high-quality *ε*-Ga_2_O_3_ (Figure 19a). The photodetector fabricated with the best crystallinity thin film has a response of 234.14 A/W and a PDCR of 10^3^, and a remarkably short 22 ms decay time.

In addition to the introduction of buffer layers, the growth of thicker films has been proved to be a means to improve epitaxial quality. Cao et al. [35] grew *ε*-Ga_2_O_3_ on *c*-Al_2_O_3_ substrate by MOCVD and investigated the effects of film thickness on the quality of the crystals. The result demonstrated that the FWHM of the film gradually narrowed down from 1.01° to 0.46°, with an increase in film thickness from 110 nm to 480 nm. They used the film with minimum FWHM of 0.46° to prepare the MSM photodetector, which illustrated an on/off ratio of over 2 × 10^3^, a high responsivity of 146 A/W at 5 V and a detectivity of 1.2 × 10^13^ Jones.

However, in general, it is difficult for high responsivity and PDCR to coexist with a fast response time; a high responsivity and a large PDCR tend to slow down the response time. As shown in Figure 19d, photodetector prepared with 480 nm *ε*-Ga_2_O_3_ has a turn-off time as high as 5.2 s. This is attributed to the photogenerated carrier in thicker film require longer drift distance for the complete depletion under the condition of the constant drift velocity of the carriers. As a result, ultrathin photodetectors tend to have extremely fast response speeds, but lose other detection indices, such as PDCR, as well as detectivity and responsivity [106]. Although it is difficult for the coexistence of above all aspects, the performance of the photodetector can be improved by the design of the electrode shapes and materials, which will be discussed in Section 4.4.

### 4.2. Oxygen Vacancy Effects

In addition to crystallinity, oxygen vacancy is commonly considered to be the key factor causing photogenerated carrier recombination in photodetectors. Reducing the concentration of oxygen vacancies in photodetectors will generally inhibit dark current with photocurrent increase. There are many methods to suppress oxygen vacancies. On one hand, the decrease in oxygen vacancy commonly occurred with crystallinity improvement. On the other hand, annealing and thickening of the film and passivation layer are also effective methods for inhibiting the oxygen vacancy’s effect on photodetectors.

Qian et al. [95] introduced an Al_2_O_3_ buffer layer to reduce the oxygen vacancy defects inside the upper epitaxial layer of *ε*-Ga_2_O_3_, enhancing the performance of the prepared photodetector in terms of dark current and transient response. Li et al. [90] determined that the oxygen vacancies were suppressed by oxidative annealing. The key parameters of the photodetector were improved by one to six orders of magnitude compared with those of pristine untreated photodetectors. The PDs, after being annealed, exhibit a PDCR of 1.06 × 10^8^, a responsivity of 1.368 A/W, and a UV–Vis suppression ratio of 1.80 × 10^7^. On the contrary, the photoelectric characteristics of the devices treated with reduction annealing are decreased. Fei et al. [96] reduced the oxygen vacancy in Ga_2_O_3_ by post-annealing, and they found that a phase transition (*ε*→*β*) occurred in the film annealed at higher temperature. The annealed film at the initial growth temperature reduced the oxygen vacancy inside the film on the premise of maintaining good crystal quality, and the photoelectric performance was improved.

**Figure 20:**
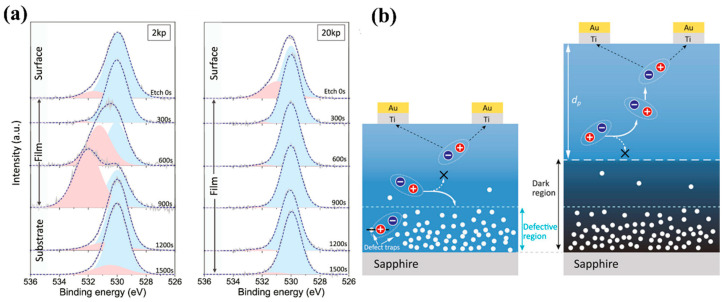
(**a**) XPS spectra of 2 kp and 20 kp thick *ε*-Ga_2_O_3_ films using the same etching depth. (**b**) Schematic diagrams of the extra dark region in assisting carrier collection in *ε*-Ga_2_O_3_ MSM thin-film photodetectors [99]. Reprinted from [99], with the permission of ACS Publications 2023.

Zhang et al. [99] grew *ε*-Ga_2_O_3_ on *c*-Al_2_O_3_ via PLD and achieved more than 18-fold enhancement of photocurrent by increasing the thickness of the *ε*-Ga_2_O_3_ layer. The thicker film yielded greater responsivity and a faster rise time. Generally, the range of the photogenerated carrier region is fixed when the same material film is illuminated with a specific light source. The defect region of the thinner film (2k pulse) will be closer to the surface (Figure 20b), which is manifested as the same XPS etching speeds will be etched into the defect region earlier (Figure 20a). Thinner films lead the photogenerated carrier region has a higher probability of overlapping with the defect region than thicker films. The defect region will trap photogenerated carriers, leading to a decrease in the photocurrent of the device. Contrastively, the top region of thicker film (20k pulse) has fewer defects to form a dark region that prevents photocarriers from flowing back, enhancing the carrier collection efficiency and photocurrent. The micrometer-thick *ε*-Ga_2_O_3_ MSM photodetector in this research has a PDCR of an astonishing 9.48 × 10^7^ at a bias voltage of 20 V, an astonishing responsivity of 1388 A/W, and a UV–Vis suppression ratio of 1.87 × 10^4^.

### 4.3. Doping Effects

For most of the semiconductor materials, doping introduces impurity energy levels, resulting in a significant change in the current. Therefore, researchers often avoid the introduction of impurities during the preparation to reduce dark current and enhance the PDCR, signal-to-noise ratio, or responsivity of the photodetectors. However, many times, the introduction of impurities is unavoidable; for example, carbon impurities will be introduced into the films via a precursor in the CVD technique, and Sn or In doping is a necessary condition for the preparation of *ε*-Ga_2_O_3_ via PVD. Contrary to convention, Sn or In tend to act as stabilizers in the *ε* phase structure in Ga_2_O_3_ rather than as N- or P-type dopers in the PVD technique, and they act more as defects to trap carriers. This is particularly important to note in the study of the effect of doping on *ε*-Ga_2_O_3_ films.

**Figure 21:**
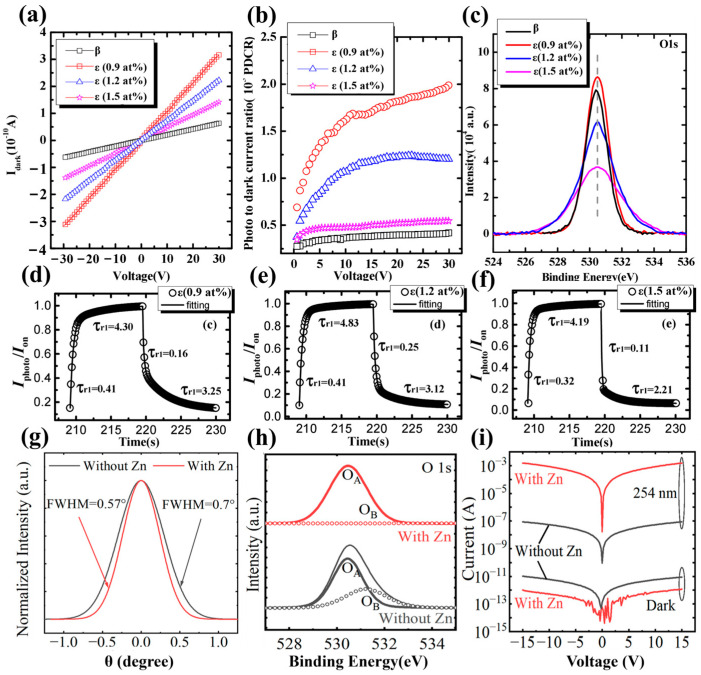
(**a**) Dark current characteristics and (**b**) characteristics of PDCR for the *β*-Ga_2_O_3_ and *ε*-Ga_2_O_3_ photodetectors; (**c**) XPS spectra of the undoped β-Ga_2_O_3_ and *ε*-Ga_2_O_3_ O1s; the current response and recovery biexponential relaxation fitting curves of (**d**) *ε*-Ga_2_O_3_ (0.9 at.% Sn), (**e**) *ε*-Ga_2_O_3_ (1.2 at.% Sn), and (**f**) *ε*-Ga_2_O_3_ (1.5 at.% Sn) [53] (reprinted from [53], with the permission of Optical Society of America 2018); (**g**) normalized XRD rocking curves of (004) plane spectra of with/without Zn-doped *ε*-Ga_2_O_3_; (**h**) high-resolution XPS core-level spectra of O1s of with/without Zn-doped *ε*-Ga_2_O_3_; (**i**) current–voltage characteristics with/without Zn-doped *ε*-Ga_2_O_3_ MSM photodetectors [92] (reprinted from [92], with the permission of RSC Publishing 2023).

Cai et al. [53] grew *ε*-Ga_2_O_3_ via PLD and investigated the effect of Sn concentration on photoelectric characteristics. At lower Sn concentrations, the film was not a pure *ε*-phase film. When the Sn doping concentration reaches 0.7 at. %, the film was composed entirely of *ε*-Ga_2_O_3_. Compared with the undoped *β*-Ga_2_O_3_, the *ε*-Ga_2_O_3_ obtained by Sn doping exhibited a relatively high dark current. With the increase in Sn content, the dark current shows a downward trend. On one hand, Sn dopants cause lattice defects which act as traps to capture carriers. With the increase in Sn doping, the effect of Sn as a defect to the trap carrier is gradually enhanced, which exacerbates the recombination effect of photogenerated carriers simultaneously, leading to a gradual decrease in the PDCR of the films with the increase in Sn content from Figure 21b. Another aspect is that the oxygen vacancies will be inhibited end up with a low dark-current through increasement of Sn shown in Figure 21c. The decay time constants *τ*_d2_ decreases from 3.80 s (*β*-) to 3.25 s (*ε*-), 3.12 s (*ε*-), and 2.21 s (*ε*-) with increase in Sn, indicating less Oxygen vacancy and other deep defects in *ε*-Ga_2_O_3_ film.

Sun et al. [92] used MOCVD to grow high-quality *ε*-Ga_2_O_3_ via introduction of Zn dopant. The films with Zn doped have a narrower FWHM and fewer oxygen vacancies compared to films without Zn as illustrated in Figure 21g,h. The addition of Zn improves crystallinity, reduces the defects of the grain boundary, and decreases the oxygen vacancies, all of which will reduce the recombination of photogenerated carriers. Meanwhile, the reduction in oxygen vacancies also inhibits the dark current. The prepared detector has a high detectivity of 1.7 × 10^16^ Jones, a large UV–Visible rejection ratio of 2.0 × 10^8^, and a fast response speed of *t*_r_ = 25 ms, *t*_d_ = 32 ms.

The above studies demonstrate that the influence of doped atoms on the film is extremely complex. In certain cases, smaller amounts of doped atoms can stabilize the phase composition and even improve the crystallinity; however, excessive doping will cause a significant decline in photoelectric properties. In general, doping that improves crystal quality is expected to enhance the photoelectric characteristics.

### 4.4. Other Factors Effect

The previous discussions have shown that the intrinsic properties of the materials have a direct impact on the performance of photodetectors. Generally, materials with higher crystal quality, less doping, and fewer oxygen vacancies have better photodetection performance. Nevertheless, this also means that the performance of the photodetectors is limited by the physical properties of the material itself. In addition to the intrinsic properties of the material, some studies illustrate that the performance of MSM photodetectors is also affected by the surface states, electrode design, and electrode materials. It has been demonstrated that surface passivation can suppress surface defects such as oxygen vacancies in photodetectors and reconstruct a carrier transport channel to enhance the response speed performance of the detector. For MSM-type photodetectors, the specific design of the electrode shape can be used to improve the specific performance. For example, narrowing the interdigital space of the electrodes can greatly improve the responsivity and detectivity of the detector, while the response speed can be accelerated by expanding interdigital space. The details are discussed in this section.

The persistent photoconductivity effect (PPC) observed in Ga_2_O_3_-based photoconductive-type devices severely affects the response time of the devices [107]. Since the occurrence of the PPC effect is related to defects in Ga_2_O_3_, especially surface defects in the form of dangling bonds or trap states [108,109,110], Zhang et al. [111] proposed passivating the defects of oxygen vacancies and Sn present on the film surface by depositing ultrathin Al_2_O_3_ as a surface passivation layer on *ε*-Ga_2_O_3_ MSM-type photodetectors. In this work, a faster surface transport channel was reconstructed to enhance the response speeds with a t_rise_ from 2.44 s to 0.182 s, a responsivity > 10^4^ A/W. This detector maintained a high level of PDCR >10^7^, as well as a UV–Vis rejection ratio greater than 10^4^.

**Figure 22:**
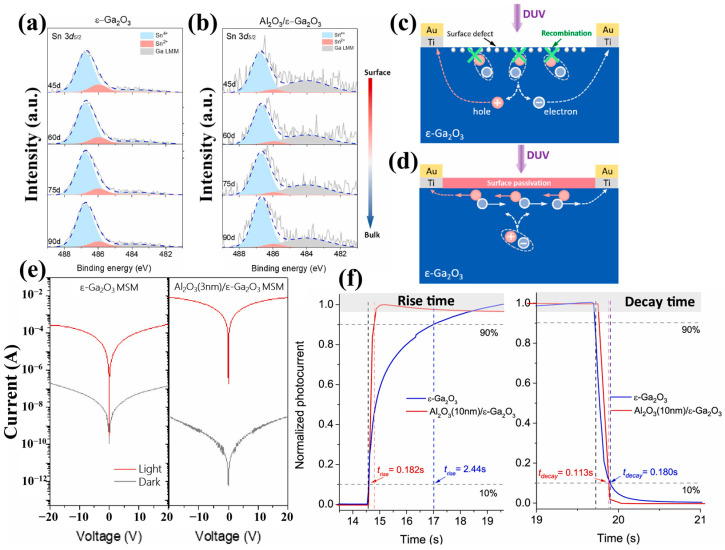
XPS spectra of the Sn 3*d* core level for (**a**) the bare *ε*-Ga_2_O_3_ film and (**b**) the Al_2_O_3_ (3 nm)/*ε*-Ga_2_O_3_ film with different collection angles; schematic of carrier transport and recombination process for (**c**) the bare *ε*-Ga_2_O_3_ photodetector and (**d**) the passivated photodetector; (**e**) the bare *ε*-Ga_2_O_3_ photodetector and the Al_2_O_3_ (3 nm)/*ε*-Ga_2_O_3_ photodetector under DUV illumination in comparison with corresponding dark currents; (**f**) rising edge and decay edge of the photocurrent for determining the rise time and the decay time [111] (reprinted from [111], with the permission of Elsevier 2023).

There are two mechanisms in improving photoelectric characteristics. Firstly, the response of the photodetectors was enhanced by rebuilding a faster surface transport channel. Secondly, as mentioned before, in the preparation of *ε*-Ga_2_O_3_ by PVD, Sn is involved as a catalyst to assist the generation of Ga_2_O_3_ (this process is mainly by inhibiting the desorption of Ga_2_O, which is oxidized to Ga_2_O_3_, and SnO_2_ is reduced to SnO). From the XPS spectrum of Figure 22a, the Sn^2+^ species is distributed both on the surface and inside region of the film, and oxygen vacancies are usually accompanied by Sn^2+^. Through introducing the Al_2_O_3_ passivation layer, the oxygen vacancies signal and the Sn^2+^ signal correlating to surface and inside are effectively suppressed (Figure 22b). As illustrated in Figure 22c,d, the passivation layer effectively prevents the recombination of photogenerated carriers, and a conductive channel was reconstructed, leading to enhancement of photocurrent and reduction in dark current, respectively (Figure 22e).

**Figure 23:**
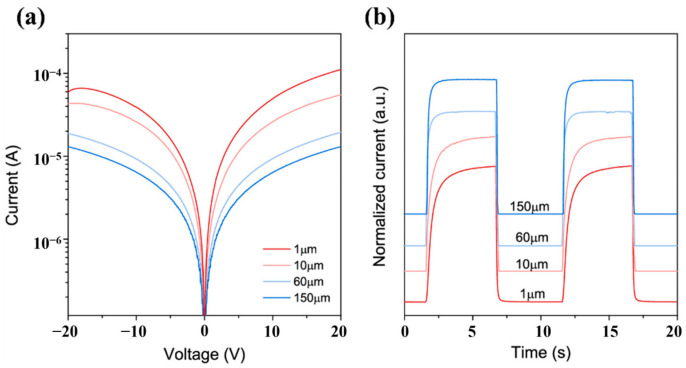
(**a**) Current–voltage and (**b**) current–time characteristics of *ε*-Ga_2_O_3_ MSM photodetectors with four different interdigital spacing of 1, 10, 60, and 150 μm [99]. Reprinted from [99], with the permission of ACS Publications 2023.

At present, most of the electrodes used in *ε*-Ga_2_O_3_-based MSM-type devices are interdigital electrodes, and the design of interdigital structure will affect the final performance of photodetector. Zhang et al. [99] designed electrodes with different spacing and tested their photoelectric performance, Figure 23 shows the photoelectric performance of *ε*-Ga_2_O_3_-based MSM-type photodetectors with different interdigital spacing. According to the current–voltage characteristics, smaller spacing will lead to higher photocurrent, which can be understood that small spacing reduces the resistance between the two electrodes.

Meanwhile, the small interdigital spacing will reduce the effective illumination area, and the light responsivity of the device will be improved consequently (the dark current will hardly change). From Figure 23b, interdigital spacing also has a greater impact on the rising response time of the detector compared to the falling response time. The smaller interdigital spacing results in a longer rising time.

Lei et al. [104] also studied the effect of interdigital spacing on the performance of the *ε*-Ga_2_O_3_-based photodetector. As spacing decreased from 25 μm to 10 μm, the photocurrent of the photodetector gradually increased, owing to reducing interdigital spacing deepening the current flow path and enhancing the extraction efficiency of both photogenerated and dark carriers in the detector. The dark current increased more than the photocurrent, leading to a decrease in PDCR, which was still in the same order of magnitude. This result provided a guide to the tradeoff between responsivity and response time when preparing photodetectors for special requirements.

In addition, electrode materials will have a significant effect on photodetectors. In traditional MSM-type photodetectors, metals such as Au, Ti, Al, and Ni are commonly used as electrode materials, and multilayer metals such as Ti/Au, Ni/Au, Cr/Au, and Ni/Al are also used to increase the wettability between the electrode and the detector material [112]. The principle of electrode selection is commonly based on forming ohmic contact with the detector material to avoid the formation of Schottky barriers that affect detector’s performance. However, metal electrodes are easily oxidized or scratched, which will gradually aggravate the performance of photodetectors, and they cannot be applied in next-generation transparent optoelectronic technologies [113]. Nonmetal materials such as high conductive F-doped and In-doped Tin oxides (FTO, ITO) and doped metal oxides such as aluminum zinc oxide and indium gallium zinc oxide (AZO, IGZO) are also recognized as suitable candidates for electrodes in UV photodetectors.

**Figure 24:**
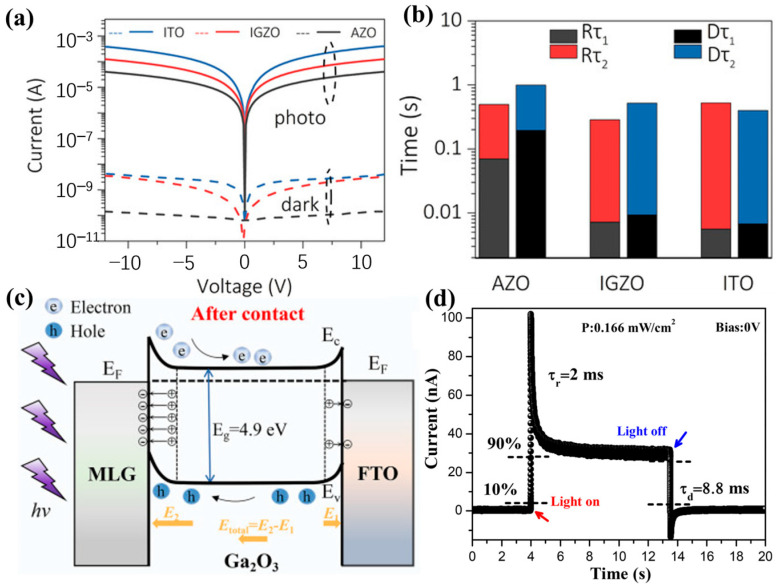
(**a**) PDCR and (**b**) photoresponse of the three kinds of photodetectors with different electronic materials [91] (reprinted from [91], with the permission of WILEY 2022). (**c**) Energy band diagram of the MLG/*β*-Ga_2_O_3_/FTO photodetector; (**d**) MLG/*β*-Ga_2_O_3_/FTO photoresponse in current towards illumination of 250 nm light [115] (reprinted from [115], with the permission of Elsevier 2023).

Zhou et al. [91] constructed *ε*-Ga_2_O_3_-based MSM-type photodetectors by using ITO, IGZO, and AZO transparent conducting oxides as electrodes. It was illustrated that the photodetectors using all three electrodes have a response rate higher than 260 A/W and an excellent detectivity greater than 10^14^. The fastest response time can be within 10 ms, as shown in Figure 24a,b. The resistivity and carrier mobility of the electrode materials are key factors affecting the performance of photodetectors. Photodetectors with the lowest resistivity ITO electrode have the highest photocurrent, responsivity, and the fastest response speed. The photodetector with AZO electrode with the highest resistivity and the lowest mobility has the lowest photocurrent performance and the slowest response time, but the highest detectivity and PDCR.

Qu et al. [114] replaced top alloy electrodes (Ti/Au) in photodetector devices by graphene, it has been demonstrated that the device fabricated by graphene electrode has a faster response time and two orders magnitude responsivity great than that of the Au/Ti top electrode device. Multilayer graphene (MLG) can be adopted as top layer electrode in *β*-Ga_2_O_3_-based solar-blind photodetectors [115]. Due to the built-in electric field generated by the dominating Schottky contact between Ga_2_O_3_ and MLG (Figure 24c), the device can be self-driven without external power supply. The device exhibited excellent photodetection performance under UV illumination at 0 V bias with a I_photocurrent max_ = 31 nA (under λ_UV_ = 250 nm), fast response speed t_rise_ = 2 ms, t_decay_ = 8.8 ms, as well as superior responsivity 9.2 mA/W (under λ_UV_ = 230 nm). Therefore, the performance of this electrode on *ε*-Ga_2_O_3_-based photodetectors is also worth exploring.

Both highly conductive MLG and transparent oxide electrodes, such as ITO, can reconstruct the energy structure between the electrode and the material to form a built-in electric field. The presence of the built-in electric field improves the mobility of photogenerated carriers, accelerates the carriers transport from the detector to the electrode, and ends up enhancing the response speed of the photodetector.

In summary, the factors affecting the performance of photodetectors are complex and coupling. The performance is mainly influenced by intrinsic properties of materials such as crystallinity, dopants, oxygen vacancies, surface state, and defects. However, certain aspects of photodetectors’ performances can also be improved by electrode shapes design and special oxide electrodes. Current research has demonstrated that high responsivity is usually accompanied with good detectivity, but researchers also note low PDCR and poor on–off speed. It is difficult to improve all property parameters simultaneously. The responsivity, PDCR, and detectivity of *ε*-Ga_2_O_3_-based photodetectors have not shown obvious advantages compared with *β*-Ga_2_O_3_. But it is easier for *ε*-Ga_2_O_3_ to obtain a faster response in similar film thicknesses. The shortest responding time has already reached to µs range. In addition, the performances of *ε*-Ga_2_O_3_ photodetectors prepared by PVD and CVD are also comparable.

### 4.5. Application Outlook

Due to its ultra-wide bandgap and large breakdown field strength, *ε*-Ga_2_O_3_ is also expected to be used in extreme environments, such as low-altitude orbit and in spacecraft [116,117,118], which challenges the irradiation resistance of *ε*-Ga_2_O_3_. Yang et al. [119] prepared *ε*-Ga_2_O_3_-based photodetectors by MOCVD and irradiated them with protons at 150 keV, with an irradiation dose of 5 × 10^15^. After irradiation, the oxygen vacancy concentration inside the film increased, the structure and light transmission performance did not change significantly, and the photodetection performance decreased slightly but still had a photo-to-dark current ratio over 10^3^, which demonstrated a great potential of *ε*-Ga_2_O_3_ in the aerospace field.

For solar-blind photodetectors, high temperature resistance is an important index. Zhang et al. [120] constructed an *ε*-Ga_2_O_3_/ZnO heterojunction self-powered photodetector and tested the stability in a high-temperature environment at 600 K. The switching time of the device did not change significantly with the temperature increase, indicating that the recombination and the generation within the photodetector were stable. In order to further enhance the thermal conductivity of *ε*-Ga_2_O_3_-based devices, Wang et al. [121] heterogeneously integrate *ε*-Ga_2_O_3_ with diamond via PLD. The *ε*-Ga_2_O_3_ grown on the diamond substrate has a high crystalline quality, and low dark current of PDs. Although the dark current increased with temperature, it remained on the order of 10^−9^, while the photo current was nearly unchanged, and the responsivity was reduced to one-third of the room temperature. *ε*-Ga_2_O_3_ is also used as a material to broaden the working range of diamond UV detectors. The detection range of conventional diamond detectors is limited below 230 nm, which cannot cover the band of 200–280 nm. Yuan et al. [101] deposited *ε*-Ga_2_O_3_ on diamond to fabricate a photodetector with composite structure. The photoelectric detection range is extended to 210 nm to 260 nm band with good signal-to-noise ratio.

## 5. Summary and Outlook

In summary, *ε*-Ga_2_O_3_ films can be fabricated via either CVD or PVD techniques. In the case of CVD, substrates play an important role as a base for *ε* phase formation. Suitable temperatures, pressures, and precursor ratios are required for *ε*-phase nucleation and pure phase growth. In the case of PVD, doping is a necessary condition for the formation of the *ε* phase. The quality of film is further affected by substrate type, deposition temperatures, and oxygen pressures. The reported photoelectrical properties of *ε*-Ga_2_O_3_-based photodetectors vary a lot. In general, defects, including grain boundaries, doping-induced lattice distortions, and oxygen vacancies, can generate trapping energy levels and obstacles for the migrations of carriers, leading to low responsivity and slow response of the *ε*-Ga_2_O_3_ films. The optimization of electrode structures and the introduction of passivation layers can strongly enhance the responsivity and detectivity of *ε*-Ga_2_O_3_ films. Special electrode materials can generate build-in electric field in *ε*-Ga_2_O_3_ films to achieve ultrafast responses. Despite the findings of current research, the formation mechanisms of *ε*-Ga_2_O_3_ films remain unclear. Especially, *ε*-Ga_2_O_3_ films without doping have never been successfully synthesized via PVD techniques. The simultaneous improvements of the photoelectrical properties (responsivity, PDCR, detectivity, responding time, etc.) of *ε*-Ga_2_O_3_ are still very difficult to attain. Moreover, many other properties and potential applications of *ε*-Ga_2_O_3_, such as power performance for electronic devices and high electron mobility properties for RF devices, are rarely investigated. It can be expected that, with further understanding of the fundamentals of *ε*-Ga_2_O_3_, the films’ properties can be enhanced significantly and specifically tuned to meet the requirements of a great range of applications.

## Figures and Tables

**Table 1:** Comparison of the intrinsic properties of Ga_2_O_3_ with other semiconductor materials.

Generation	First	Second		Third		
Materials	Si	GaAs	InP	4H-SiC	GaN	Ga_2_O_3_
Band gap (eV)	1.12	1.43	1.3	3.3	3.4	4.7–5.2
Relative permittivity	11.7	12.9	12.5	9.7	9	10
Dielectric strength (MV/cm)	0.3	0.4	0.5	2.5	3.3	8
Heat conductivity (W/cm·K)	1.5	0.05	0.7	4.8	2.1	0.23 [010] 0.13 [100]
Electron mobility (cm^2^/V·s)	1400	8500	5400	1000	1200	300
Baliga’s figure of merit (eμEc^3^)	1	14.7	/	317	846	3214

**Table 2:** Depositions of Ga_2_O_3_ by CVD methods.

MOCVD				
Substrate	Precursor	Deposition Conditions	Phase and Properties	Ref.
*c*-Al_2_O_3_	TEGa: 22.3–67.4 μmol/min O_2_: 13.4–120.5 mmol/min Carried by Ar	Pressure: 9.1 Torr Temperature: 450–570 °C	*ε*-Ga_2_O_3_	[34]
*c*-Al_2_O_3_	TEGa: 33 sccm O_2_: 47.7 sccm Carried by N_2_	Pressure: 150 Torr Temperature: 500 °C	*ε*-Ga_2_O_3_ FWHM: 0.46°	[35]
*c*-Al_2_O_3_	TEGa: 15 μmol/min H_2_O: 11 mmol/min Carried by Ar	Step1: nucleation growth with 550 °C and 100 Torr Step2: epilayer growth with 640 °C and 20 Torr	*ε*-Ga_2_O_3_ Thickness: 1.5 μm	[36]
AlN Si (111)	[H_2_O]/[TEGa] [O_2_]/[TEGa] molar ratio: 69–137/103 Carried by Ar	Step1: nucleation growth with 550–560 °C and 60~80 Torr VI/III: 69–137 Step2: epilayer growth with 640 °C and 20–28 Torr VI/III: 103	*ε*-Ga_2_O_3_ Thickness: 360 nm	[37]
*c*-Al_2_O_3_/ GaN(111)/ 3C-SiC (001)	[H_2_O]/[TMG] pressure ratio: 102–103 Carried by H_2_	Pressure: 100 mbar Temperature: 450–715 °C	*ε*-Ga_2_O_3_ Ferroelectricity Stability boundaries Band gap: 4.6–4.7 eV N-type doping	[31,38]
				[21]
				[39]
				[40]
*c*-Al_2_O_3_	[H_2_O]/[TMGa]/[TMIn]/[SiH_4_] TEGa: 5 sccm H_2_O: 1600 sccm Carried by H_2_	Pressure: 50 mbar Temperature: 610–690 °C	*ε*/*β*-Ga_2_O_3_ Phase Variation Thermal stability High conductivity by In doped	[22,41]
				[42,43]
*c*-Al_2_O_3_	[O_2_]/[TEGa] [O_2_]/[TEGa] molar ratio: 390 Flow rate of HCL: 0/5/10/30/60 sccm Carried by Ar	Pressure: 45 Torr Temperature: 600 °C Phase Controlled by changing the HCL flow	Solar-blind photodetector with a low dark current and a high PDCR	[44,45]
**Mist-CVD**				
**Substrate**	**Precursor**	**Deposition Conditions**	**Properties**	**Ref.**
NiO(111)	GaCl_3_ and C_10_H_14_NiO_4_ in deionized water Carrier gas and flow rate: N_2_: 9 L/min Molar concentrations of the Ga and Ni: 0.5 M/0.03 M	Temperature: 400–800 °C Step1: growth NiO buffer layers with N_2_ 5 L/min Step2: growth*ε*-Ga_2_O_3_ layers with N_2_ 9 L/min	*ε*/*α*-Ga_2_O_3_ Band gap: 4.9–5.3 eV RMS roughness: 1.7 nm at 750 °C	[46]
*c*-Al_2_O_3_/ GaN on sapphire/ GGG (111)	(Ga(acac)_3_) and SnCl_2_–2H_2_O in deionized water Carrier gas and flow rate: N_2_: 5 L/min Molar concentrations of the Ga: 0.05 M	Temperature: 670–700 °C	*ε*/*α*-Ga_2_O_3_ Phase stability by Sn Epitaxial relationship	[47,48]
c-Al_2_O_3_/ MgO (111)/ YSZ (111)/ AlN (0001)	(Ga(acac)_3_)/GaCl_3_ with InCl_3_/SnCl_4_/(Al(acac)_3_) Carrier gas and flow rate: N_2_: 3–9 L/min: Molar concentrations of the Ga: 0.03–0.5 M	Temperature: 400–800 °C Al doping, In doping and Sn doping	*ε*-Ga_2_O_3_ Tunable bandgap: 4.5–5.0 eV (In-doped) 5.0–5.9 eV (Al-doped) Lowest FWHM:0.31° Ferroelectric property	[49]
				[50]
				[51]
				[52]

**Table 3:** Depositions of Ga_2_O_3_ by PVD methods.

PLD				
Substrate	Target	Deposition Conditions	Properties	Ref.
*c*-Al_2_O_3_	Sn atomic percent of 0.3–1.5% Sn-doped Ga_2_O_3_	Temperature: 650 °C Laser frequency: 3 Hz (Growth for 6000 pluses) Energy density: 2.0 J/cm^2^ Oxygen pressure: 0.01 mbar target-to-substrate distance: not mention	Improved I_photo_ and R in comparison with *β*-Ga_2_O_3_ Band gap: 4.81–4.94 eV	[53]
*c*-Al_2_O_3_/ YSZ (111)/ SrTiO_3_(111)/ MgO (111)	1 wt% SnO_2_-doped Ga_2_O_3_ Al doping, In doping and Sn doping	Temperature: 410–670 °C Laser frequency: 1 Hz for NL (Growth for 300 pulses) 10 Hz for film (Growth of 15,000 pulses) Energy density: 2.0 J/cm^2^ Oxygen pressure: 3 × 10^−4^–1 × 10^−2^ mbar target-to-substrate distance: not mention	*ε*-Ga_2_O_3_ Phase stability by Sn Band gap: 4.1–4.9 eV (In-doped) 4.9–5.8 eV (Al-doped)	[54] [55] [56] [57] [58]
(0001) GaN on *c*-Al_2_O_3_	1 at% SnO_2_ and 1.5 at% ZrO_2_ doped Ga_2_O_3_	Temperature: 500/570 °C Laser frequency: 3 Hz (Growth for 5000/6000 pluses) Energy density: 2.0 J/cm^2^ Oxygen pressure: 5 × 10^−3^–6 × 10^−2^ mbar target-to-substrate distance: 50 mm [59]; not mention [60]	Resistivity: 2.4 Ω⋅cm Carrier concentration: 1.6 × 10^17^/cm^3^ Mobility: 17.4 cm^2^/Vs	[59]
				[60]
*c*-Al_2_O_3_	1% Sn-doped Ga_2_O_3_	Temperature: 800 °C Energy density: 2.0 J/cm^2^ Oxygen pressure: 200 mTorr target-to-substrate distance: 55 mm;	PDs Responsivity: 52.77 A/W at 240 nm EQE: 2.7 × 10^4^% Dark current: 5.5 × 10^−11^ A UV–vis rejection ratio: 1.2 × 10^4^	[61]
*a/c*-Al_2_O_3_	1 mol% Sn-doped Ga_2_O_3_	Temperature: 600–800 °C Laser frequency: 5 Hz (Growth for 5000/6000 pluses) Energy density: 0.6–2.5 J/cm^2^ Oxygen pressure: 0.1–200 mTorr target-to-substrate distance: 50 mm;	*α/ε*/*β*-Ga_2_O_3_	[62]
**MBE**				
**Substrate**	**Source**	**Deposition Conditions**	**Properties**	**Ref.**
*α*-Al_2_O_3_ (0001)	BEP_Ga_: 3.4 × 10^−7^ mbar, BEP_In_: 1.2 × 10^−7^ mbar, O_2_: 1 sccm	Temperature: 590–700 °C plasma power: 150–225 W In doped: 0.7–15%	*ε*/*β*-Ga_2_O_3_	[63]
*c*-Al_2_O_3_ (0001)	BEP_Ga_: 2.1 × 10^−8^–2.74 × 10^−7^ mbar BEP_Sn_: 0–1.46 × 10^−9^ mbar O_2_: 0.5 sccm	Temperature: 700 °C plasma power: 150 W	*ε*/*β*-Ga_2_O_3_	[64]

**Table 4:** The lattice mismatch between *ε*-Ga_2_O_3_ and substrates with specific matching relationships.

Substrate	Lattice Matching Relationships	Mismatch (%)
*α*-Al_2_O_3_ (0001)	$(10\overset{-}{1}$0) _film_//$(11\overset{-}{2}$0) _sub_	4.1 [31]
GaN (0001)	$(10\overset{-}{1}$0) _film_//$(11\overset{-}{2}$0) _sub_	2.55
AlN (0001)	$(10\overset{-}{1}$0) _film_//$(11\overset{-}{2}$0) _sub_	1.95
6H-SiC (0001)	$(10\overset{-}{1}$0) _film_//$(11\overset{-}{2}$0) _sub_	5.31
4H-SiC (0001)	$(10\overset{-}{1}$0) _film_//$(11\overset{-}{2}$0) _sub_	5.61
YSZ (111)	$(11\overset{-}{2}$0) _film_//(011) _sub_	19.88 [54]
STO 111)	$(11\overset{-}{2}$0) _film_//(011) _sub_	5.05 [54]
MgO (111)	$(11\overset{-}{2}$0) _film_//(011) _sub_	2.61 [54]

**Table 5:** Comparison of key parameters of MSM-type *ε*-Ga_2_O_3_ solar-blind PDs.

Phase	Methods	PDCR	R (A/W)	D* (Jones)	τ_r_ (s)	τ_d_ (s)	Bias (V)	Ref.
*ε*-Ga_2_O_3_	MOCVD	1.0 × 10^3^	0.38	6.3 × 10^10^	0.55	0.58/3.14	15	[78]
*ε*-Ga_2_O_3_	MOCVD	—	—	—	2.00	0.40/2.00	10	[39]
*ε*-Ga_2_O_3_	MOCVD	2.0 × 10^3^	146	1.2 × 10^13^	7.60	5.20	5	[35]
*ε*-Ga_2_O_3_	MOCVD	5.7 × 10^4^	84	4.2 × 10^14^	—	0.10	6	[44]
*ε*-Ga_2_O_3_	MOCVD	1.06 × 10^8^	1.368	9.13 ×10^14^	0.061	0.087	5	[90]
*ε*-Ga_2_O_3_	MOCVD	5.7 × 10^4^	84	4.2 × 10^14^	2	0.1	6	[44]
*ε*-Ga_2_O_3_	MOCVD	10^3^	146	1.2 ×10^13^	—	5.2	5	[35]
*ε*-Ga_2_O_3_	MOCVD	10^5^	286	10^14^	0.0056	0.0072	10	[91]
*ε*-Ga_2_O_3_	MOCVD	10^8^	—	1.7 × 10^16^	0.025	0.032	10	[92]
*ε*-Ga_2_O_3_	MOCVD	10^5^	230	1.2 × 10^15^	—	0.024	6	[93]
*ε*-Ga_2_O_3_	MOCVD	10^3^	243	1.83 × 10^17^	—	0.022	10	[94]
*ε*-Ga_2_O_3_	MOCVD	10^4^	8.1	10^13^	0.87	0.29	10	[95]
*ε*-Ga_2_O_3_	MOCVD	3 × 10^5^	1000	1.0 × 10^15^	—	0.03 s/0.11	10	[96]
*ε*-Ga_2_O_3_	RF Sputter	1.68 × 10^2^	0.077	2.85 × 10^12^	1.6/7.4	5.4/0.67	5	[97]
*ε*-Ga_2_O_3_	RF Sputter	1.45 × 10^5^	6.18	5 × 10^13^	0.14	0.09	10	[98]
*ε*-Ga_2_O_3_	PLD	9.48 × 10^7^	1388	—	0.166	0.075	20	[99]
*ε*-Ga_2_O_3_	PLD	1.0 × 10^5^	3.74	—	0.41/4.30	0.16/3.25	20	[53]
*ε*-Ga_2_O_3_	PLD	10^4^	506	—	—	0.04	15	[100]
*ε*-Ga_2_O_3_	PLD	5.7 × 10^4^	—	—	0.64	0.13	30	[101]
*β*-Ga_2_O_3_	MBE	1.0 × 10^4^	259	—	2.10	0.40	20	[102]
*β*-Ga_2_O_3_	Mist-CVD	1.5 × 10^5^	22,000	8.0 × 10^16^	0.51/2.76	0.21/1.38	20	[103]
*ε*-Ga_2_O_3_	Mist-CVD	6.7 × 10^5^	0.085	3.86 × 10^12^	0.191	0.545	20	[104]
α/*β*-Ga_2_O_3_	Sol-Gel	1.6 × 10^3^	0.041	5.41 × 10^11^	0.03/0.23	0.04/0.41	15	[105]

## Data Availability

No new data were created or analyzed in this study.
